# Stapled Phd Peptides Inhibit Doc Toxin Induced Growth Arrest in *Salmonella*

**DOI:** 10.1021/acschembio.3c00411

**Published:** 2023-11-21

**Authors:** Dennis J. Worm, Grzegorz J. Grabe, Guilherme V. de Castro, Sofya Rabinovich, Ian Warm, Kira Isherwood, Sophie Helaine, Anna Barnard

**Affiliations:** † Department of Chemistry, Molecular Sciences Research Hub, Imperial College London, 82 Wood Lane, London W12 0BZ, U.K.; ‡ Department of Microbiology, Harvard Medical School, 4 Blackfan Circle, Boston, Massachusetts 02115, United States

## Abstract

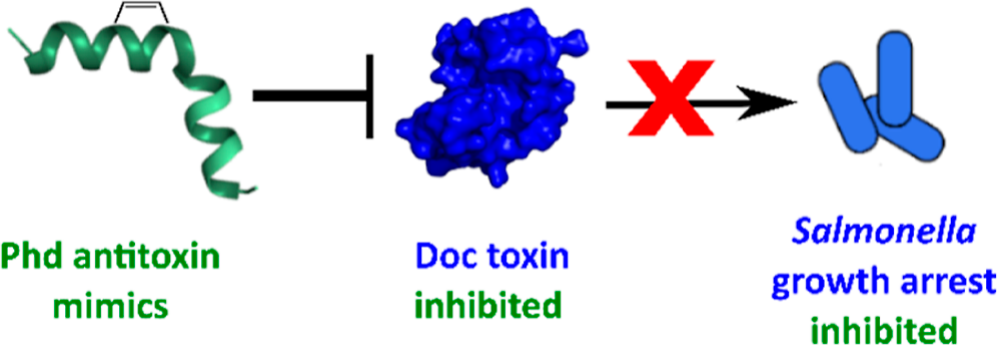

Bacterial toxin inhibition is a promising approach to overcoming antibiotic failure. In*Salmonella*, knockout of the toxin Doc has been shown to significantly reduce the formation of antibiotic-tolerant persisters. Doc is a kinase that is inhibited in nontolerant cells by its cognate antitoxin, Phd. In this work, we have developed first-in-class stapled peptide antitoxin mimetics based on the Doc inhibitory sequence of Phd. After making a series of substitutions to improve bacterial uptake, we identified a lead stapled Phd peptide that is able to counteract Doc toxicity in *Salmonella*. This provides an exciting starting point for the further development of therapeutic peptides capable of reducing antibiotic persistence in pathogenic bacteria.

## Introduction

Protein–protein interactions (PPIs) are increasingly recognized as tractable drug targets, despite their initial “undruggable” reputation.^1,2^ Over recent years, a number of strategies have emerged for their successful modulation, with peptide “stapling” becoming a frequent method of choice.^3,4^ “Stapled” peptides are derived from the binding sequence of one protein partner of an α-helix-mediated PPI. Outside of the stabilizing environment of a whole protein, peptide sequences may not adopt a well-defined helical conformation. This results in susceptibility to proteolytic degradation and, often, limited cell uptake.^5^ To circumvent these issues, amino acids capable of chemical cross-linking to one another can be introduced into the active sequence at a spacing of one (*i*, *i* + 4) or multiple (*i*, *i* + 7 or *i*, *i* + 11) helical turns.^6^ The formation of a covalent bond, a “staple”, between these two residues constrains the peptide into a permanent helical conformation, improving affinity, stability, and cell penetration,^7^ including in Gram-negative bacteria.^8,9^ Since their initial introduction, stapled peptides have risen in prominence, providing validated inhibitors for multiple targets in cancer,^10,11^ including some progressing to clinical trials.^12,13^ Beyond cancer, stapled peptides have also shown promise in malaria^14^ and for bacterial targets, with a number of examples with efficacy against drug transporters,^15^ cell division,^16^ and gene transcription.^8^ In addition, stapling has been shown to improve the activity and stability of antimicrobial peptides.^17−21^

Bacterial toxins are a class of ubiquitous proteins which act in response to stress and represent an untapped pool of targets for inhibition by peptide ligands.^22^ Toxins inhibit key cellular processes, leading to bacterial growth arrest, and have been linked to increased survival of bacteria to host immune defense, antibiotic treatment, and bacteriophages.^23,24^ They are expressed alongside a cognate antitoxin, which, for Type II toxin-antitoxin systems, forms an inhibitory PPI in nonstressed cells.^25^ Activation of the toxin through degradation or reduced expression of the antitoxin results in bacterial growth arrest, enabling survival under stress conditions.^26^ Therefore, toxin inhibition, through mimicry of the mode of action of the antitoxin, could provide a mechanism to reduce population survival under antibiotic stress.^27,28^

In *Salmonella enterica* serovar typhimurium (*S. typhimurium*), growth-arrested antibiotic persisters are formed upon macrophage internalization and complicate clearance of the infection. The number of macrophage-induced antibiotic-tolerant cells is significantly reduced upon knockout of the *phd-doc* toxin-antitoxin module encoding for the toxin Doc and its antitoxin partner, Phd.^23^ Doc functions as a kinase, phosphorylating the translation elongation factor EF-Tu and subsequently inhibiting protein synthesis.^29,30^ We have previously carried out a comprehensive characterization of the Phd-Doc interface and demonstrated that antitoxin peptides can effectively mimic the activity of the full length Phd protein both in vitro and when expressed in *S. typhimurium*.^31^

**Figure 1:**
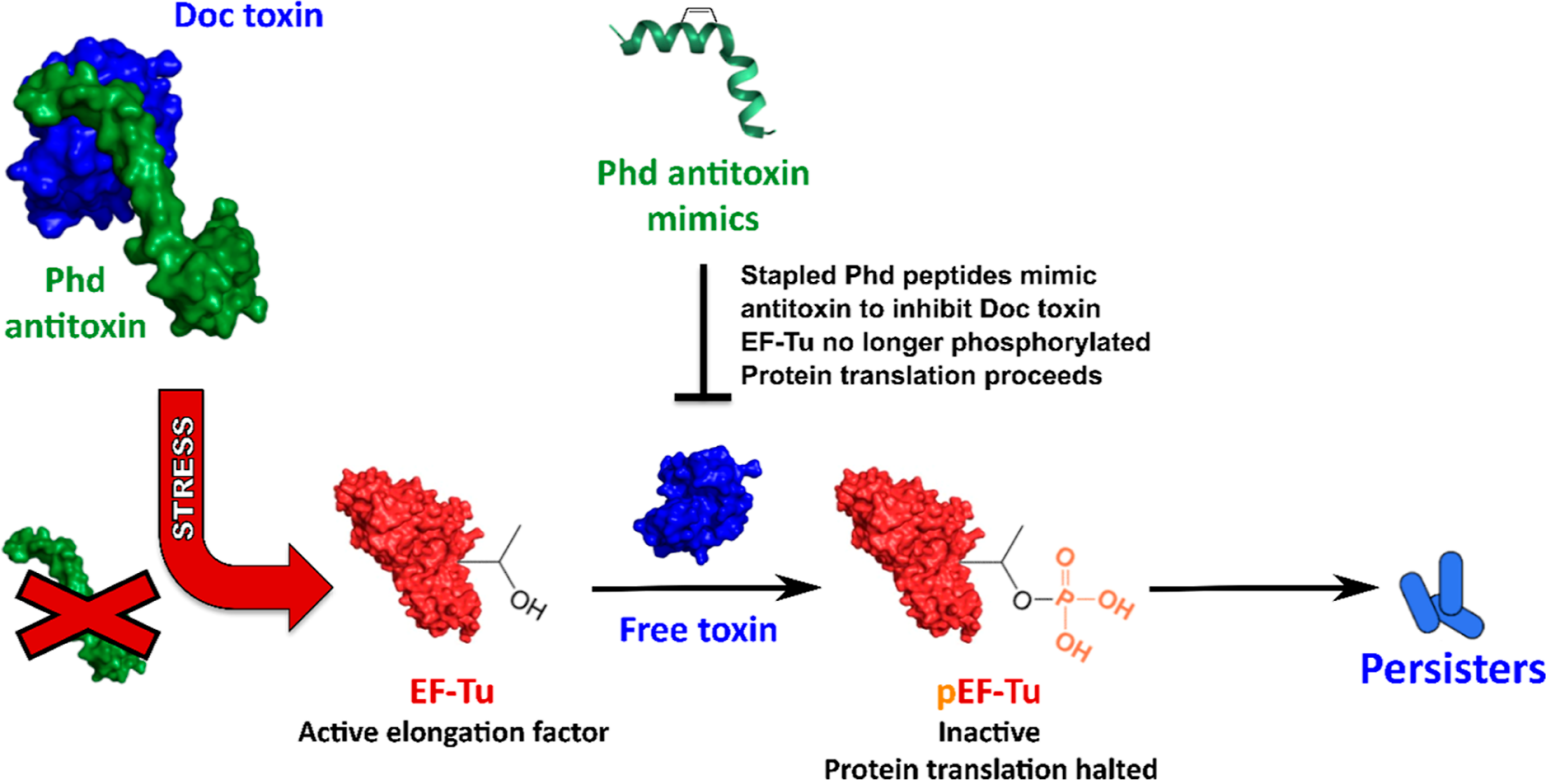
Under stress, Phd is degraded or no longer expressed releasing free Doc toxin which phosphorylates EF-Tu, resulting in *Salmonella* growth arrest and persister formation. Stapled Phd peptides mimic the Doc-binding domain of the antitoxin, inhibiting Doc and preventing persister formation.

Here, we report the development of stapled Phd peptides capable of the rescue of Doc-induced growth arrest when administered to *S. typhimurium* (Figure 1). Using the C-terminus from *S. typhimurium* Phd (Phd_STm_^52–73^) with pM affinity for Doc (Doc_STm_) as a template, we generated a library of analogues. A combination of residue substitutions and hydrocarbon stables was used to reduce the overall negative charge of the sequence and enhance bacterial uptake while minimizing negative effects on affinity and Doc_STm_ inhibition activity. *S. typhimurium* cultures were then treated with a subset of optimized peptides, which proved capable of counteracting the effects of Doc_STm_ toxicity. This study provides the first example of extracellularly administered inhibitors of toxin-induced growth arrest and promising starting points for the optimization of stapled peptide toxin inhibitors as agents to reduce antibiotic persistence.

## Results

### Arginine Scan of Phd^52–73^ Peptide to Reduce the Negative Charge of the Wild-Type Sequence

**Figure 2:**
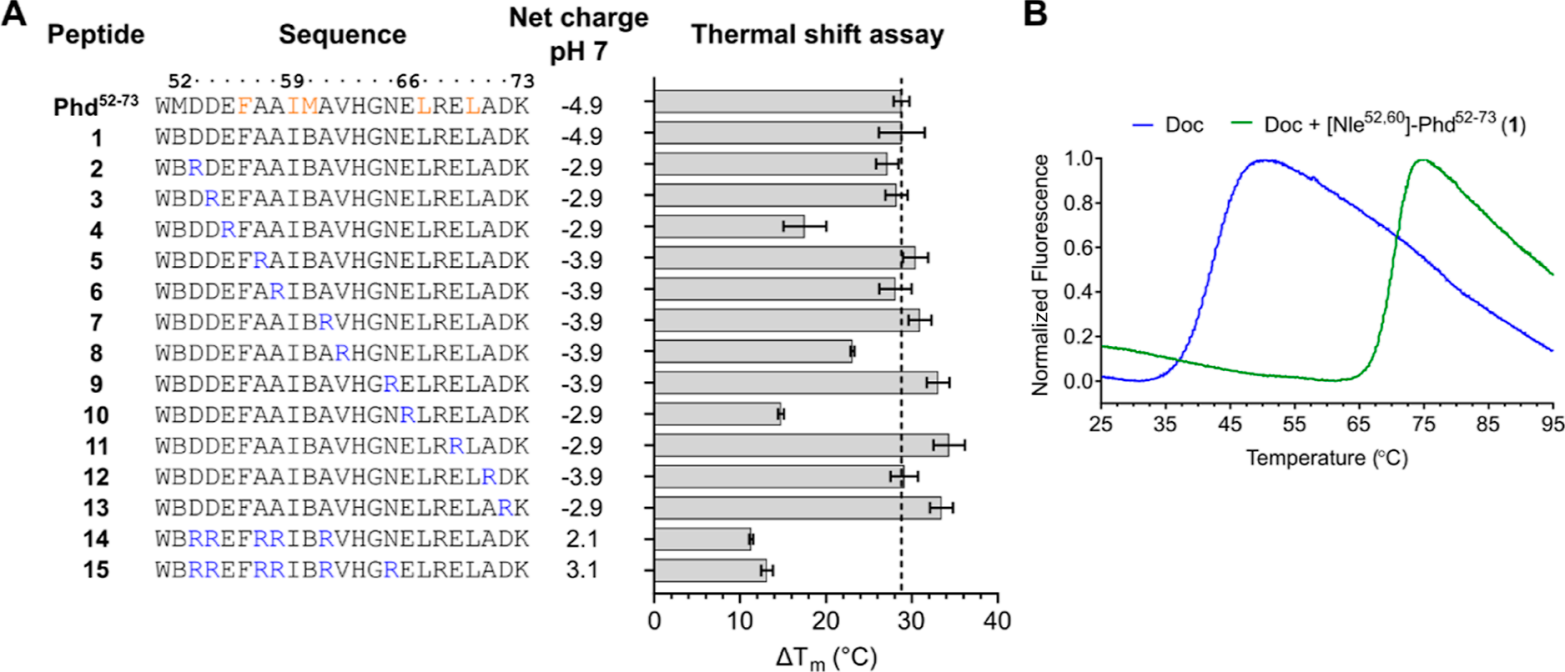
Arginine scan of Phd^52–73^ peptide analogue 1. (A) Peptide sequences, peptide net charge at pH 7, and Δ*T*_m_ (thermal shift assay) values of the interaction of Doc_STm_ to peptides Phd^52–73^, **1** and arginine-scan analogues **2**–**15**. Hot-spot residues important for Doc binding and inhibition are marked in orange in the Phd^52–73^ peptide. Δ*T*_m_ values are shown as the mean ± SD. B = l-norleucine. (B) Thermal shift denaturation curves of free Doc_STm_ at 5 μM (blue) and in the presence of 50 μM Phd^52–73^ peptide **1** (green). The sigmoidal sections selected for the melting temperature fit are shown in black.

We previously characterized the C-terminal domain of *S. typhimurium* Phd antitoxin peptide (Phd_STm_^52–73^) as a high affinity inhibitor of Doc_STm_ toxin, exhibiting pM binding affinity and Doc_STm_ inhibition activity comparable to full-length Phd_STm_^1–73^ protein.^31^ Based on this activity, the Phd_STm_^52–73^ peptide holds great promise as a Doc_STm_ toxin inhibitor to tackle and study antibiotic persistence in *S. typhimurium*. However, due to its high negative net charge of −4.9 at neutral pH 7 (Figure 2A), it was expected that the Phd_STm_^52–73^ peptide would exhibit very low penetration into Gram-negative bacteria, necessitating the development of a cell-permeable Phd_STm_^52–73^ variant able to target the intracellular toxin.

As a starting point for the generation of cell-permeable Phd_STm_^52–73^ peptides, we used the wild-type Phd^52–73^ sequence with N-terminal acetylation and an additional N-terminal tryptophan residue to allow spectrophotometric quantification, as previously described.^31^ All peptides were prepared by an automated microwave-assisted solid-phase peptide synthesis (SPPS) using the Fmoc/*t*-Bu strategy. As an initial modification, Met52 and Met60 of Phd^52–73^ were replaced with norleucine to prevent oxidation problems and interference with hydrocarbon stapling, resulting in peptide **1** (Figure 2A). The binding of peptide **1** to recombinantly produced Doc_STm_ was verified by a thermal shift assay (Figure 2A,B), revealing a large thermal stabilization of Doc_STm_ by **1** with a positive melting temperature (*T*_m_) shift of 28.8 ± 2.7 °C. The observed thermal shift was the same as previously measured for wild-type Phd^52–73^ (Δ*T*_m_ = 28.8 ± 0.9 °C), confirming that methionine replacement by norleucine did not alter the Doc_STm_ binding affinity of the peptide. Therefore, peptide **1** was used as a template for the generation of further Phd^52–73^ analogues.

In a first development cycle toward cell-permeable Phd_STm_^52–73^ peptides, we performed an arginine scan of **1** to identify residues suitable for the introduction of positive charges without loss of Doc_STm_ binding affinity. Peptide variants with single arginine substitutions of all nonarginine residues except for the previously identified hot-spot residues for Doc_STm_ binding and inhibition (Phe56, Ile59, Nle60, His63, Leu67, and Leu70),^31^ as well as Nle52 and Lys73, were prepared (**2**–**13**, Figure 2A). Thermal shift analysis of Doc_STm_ in the presence of each peptide revealed that only the substitution of Glu55, Val62, and Glu66 was not fully tolerated, as displayed by the reduced thermal stabilization of Doc_STm_ for these peptides (Δ*T*_m_ ≈ 15–23 °C).

To check the effect of multiple arginine substitutions on the Doc_STm_ binding of **1**, peptides **14** and **15** with five and six well-tolerated arginine substitutions, respectively, were synthesized. However, the introduction of this larger number of arginine residues in **14** and **15** led to a significant loss of Doc_STm_ stabilization (Δ*T*_m_ ≈ 11–13 °C).

### N-Terminal Arginine-Spiking and Stapling Retain In Vitro Toxin Inhibition and Enable Bacterial Uptake

Since the introduction of a larger number of arginine residues in peptide **1** proved to be detrimental to Doc_STm_ binding and single arginine substitutions are unlikely to be sufficient to improve bacterial uptake, we investigated if the substitution of a few selected residues by arginine in combination with *i*, *i* + 4 hydrocarbon stapling would generate Phd^52–73^ analogues with reduced negative charge and simultaneous staple-induced improved bacterial penetration. Pentenylalanine residues stapled using Grubbs metathesis were selected due to their prevalence in the field and ease of synthesis.^32^

**Figure 3:**
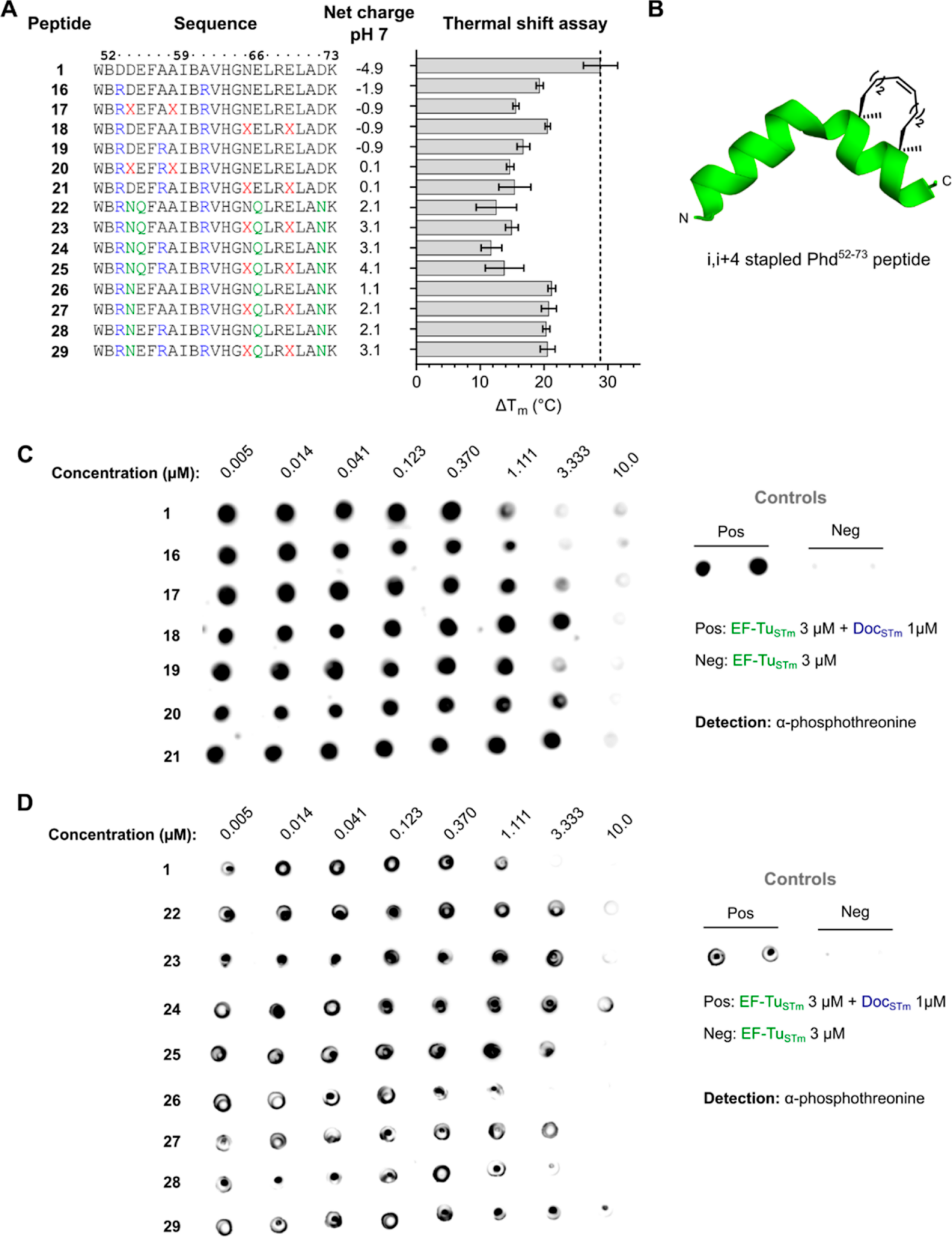
N-terminally arginine-spiked and hydrocarbon-stapled Phd^52–73^ peptides with in vitro Doc inhibitory activity. (A) Peptide sequences, peptide net charge at pH 7, and Δ*T*_m_ (thermal shift assay) values of the interaction of Doc_STm_ to Phd^52–73^ peptide **1** and analogues **16**–**29**. Δ*T*_m_ values are shown as mean ± SD. B = l-norleucine, X = (*S*)-2-(4-pentenyl)-alanine. (B) Schematic depiction of C-terminally *i*, *i* + 4 hydrocarbon-stapled Phd^52–73^ peptide. The shown peptide structure is derived from the homology model of Doc_STm_ bound to Phd_STm_^52–73^, as previously described. (C) Dot blot detection of phosphorylated EF-Tu_STm_ in the presence of Doc_STm_ and Phd^52–73^ peptides **1** and **16**–**21**. Peptides were tested at eight concentrations, ranging from 10 μM to 5 nM (3-fold dilutions). Negative (EF-Tu_STm_ 3 μM) and positive (EF-Tu_STm_ 3 μM + Doc_STm_ 1 μM) phosphorylation controls of the assay are shown on the right. (D) Dot blot detection of phosphorylated EF-Tu_STm_ in the presence of Doc_STm_ and Phd^52–73^ peptides **1** and **22**–**29**. Assay controls are shown on the right.

Residues Asp53 and Ala61 or Asp53, Ala57, and Ala61 in the N-terminal part of peptide **1** were substituted for arginine to generate analogues **16** and 1**9** with two or three additional arginines and net charges of −1.9 and −0.9, respectively (Figure 3A). These substitutions were combined with *i*, *i* + 4 hydrocarbon stapling between residues 4 and 8 in the N-terminal half of the peptide (**17** and **20**) as well as stapling between residues 15–19 in the C-terminal half of the peptide (**18** and **21**) to obtain analogues with net charges of −0.9 to 0.1 (Figure 3A,B). For modified peptides, **16**–**21** a loss in Doc_STm_ stabilization compared to **1** was observed by thermal shift (Δ*T*_m_ ≈ 15–20 °C). In addition, the Doc inhibition activity of the peptide analogues was assessed in a phosphorylation assay with recombinant EF-Tu_STm_ and Doc_STm_ and analyzed by dot blot as previously described.^31^ Peptide **1** and analogues **16** and **19** fully inhibited EF-Tu_STm_ phosphorylation by Doc_STm_ when present at three times the concentration of Doc_STm_ in the assay (1 μM), while analogues **17**, **18**, **20,** and **21** required a higher concentration to achieve full inhibition (Figure 3C).

As the net charge of peptides **16**–**21** at neutral pH was still negative or zero, charge reversal of the peptide sequence by additional removal of negatively charged aspartic acid and glutamic acid residues was investigated. Arginine-spiked peptide sequences **16** and **19** were further modified by substitution of Asp54 and Asp72 with asparagine and substitution of Glu55 and Glu66 with glutamine to obtain analogues **22** and **24** with positive net charges of 2.1 and 3.1, respectively. This was additionally combined with *i*, *i* + 4 hydrocarbon stapling between residues 15–19 in the C-terminal half of the peptide to gain the positively charged stapled analogues **23** and **25** (Figure 3A). Peptides **22**–**25** still bound to Doc_STm_ but exhibited a further decreased stabilization compared to **16**–**21** by thermal shift (Δ*T*_m_ of **22**–**25** ≈ 12–15 °C). Furthermore, a concentration of **22**–**25** higher than three times the Doc_STm_ concentration was required for full Doc_STm_ inhibition in the EF-Tu_STm_ phosphorylation assay (Figure 3D). Due to the very high evolutionary conservation of Glu55 in Phd antitoxin across bacterial species and the significant loss in Doc interaction when replacing this residue with arginine (peptide **4**, Figure 2A), we speculated that substitution of Glu55 for glutamine might have caused the observed loss in Doc binding affinity in peptides **22**–**25**. Accordingly, we prepared analogues **26**–**29**, which replicate **22**–**25** but still contain residue Glu55, resulting in peptides with positive net charges of 1.1–3.1 (Figure 3A). The presence of Glu55 was sufficient to regain Doc_STm_ binding affinity, as analogues **26**–**29** demonstrated a significantly higher thermal stabilization of Doc_STm_ in the thermal shift assay (Δ*T*_m_ of **26**–**29** ≈ 20–21 °C). In addition, the unstapled peptides **26** and **28** demonstrated a Doc_STm_ inhibition activity similar to that of **1** in the EF-Tu_STm_ phosphorylation assay (Figure 3D), while the stapled peptides **27** and **29** still required a higher concentration for full Doc_STm_ inhibition.

**Figure 4:**
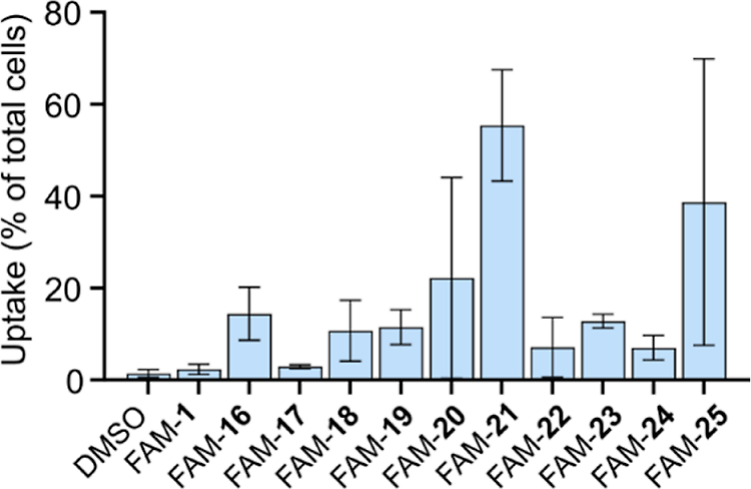
Bacterial uptake of first series of Phd^52–73^ analogues in *E. coli*. Cellular uptake of 5 μM fluorescein (FAM)-labeled Phd^52–73^ peptides **16**–**25** in *E. coli* MG1655 after incubation for 2 h at 37 °C as determined by flow cytometry. Data are shown as mean ± SD. Precipitation was observed for peptides FAM-**20**, FAM-**21,** and FAM-**25** in the assay.

Peptide analogues **16**–**25** as well as base peptide **1** were subsequently analyzed for their cellular uptake into *E. coli* as a Gram-negative model bacterium, to check if the introduced sequence modifications and stapling translated into a higher bacterial cell penetration of the Phd^52–73^ peptide. For the uptake studies, *E. coli* MG1655 cells were treated with 5 μM of fluorescein (FAM)-labeled peptide variants for 2 h, and the penetration of the peptides into the bacterial cells was determined by flow cytometry. As expected, the highly negatively charged peptide FAM-**1** displayed extremely low uptake into *E. coli*, while FAM-labeled **16**, **18**, **19,** and **22**–**24** exhibited an increased bacterial uptake with penetration efficiencies of around 7–14% (Figure 4). For the FAM-labeled versions of stapled **20**, **21,** and **25**, precipitation in the assay conditions was observed, making result interpretation difficult due to large errors and despite the use of trypan blue as an extracellular FAM quencher in the assay. Nevertheless, these first uptake studies demonstrated that the applied sequence modifications yielded a higher uptake of the Phd^52–73^ peptide into Gram-negative *E. coli*.

### N-Terminally Hydrocarbon-Stapled and C-Terminally Arginine-Spiked Phd^52–73^ Peptides with In Vitro Doc_STm_ Inhibition Activity

Since arginine substitutions in the C-terminal half of Phd^52–73^ peptide **1** were, apart from Glu66Arg, also well tolerated, we additionally explored arginine-spiking toward the C-terminus of **1** combined with acidic residue removal and N-terminal hydrocarbon stapling for the generation of positively charged and potentially cell-permeable Phd^52–73^ peptides.

**Figure 5:**
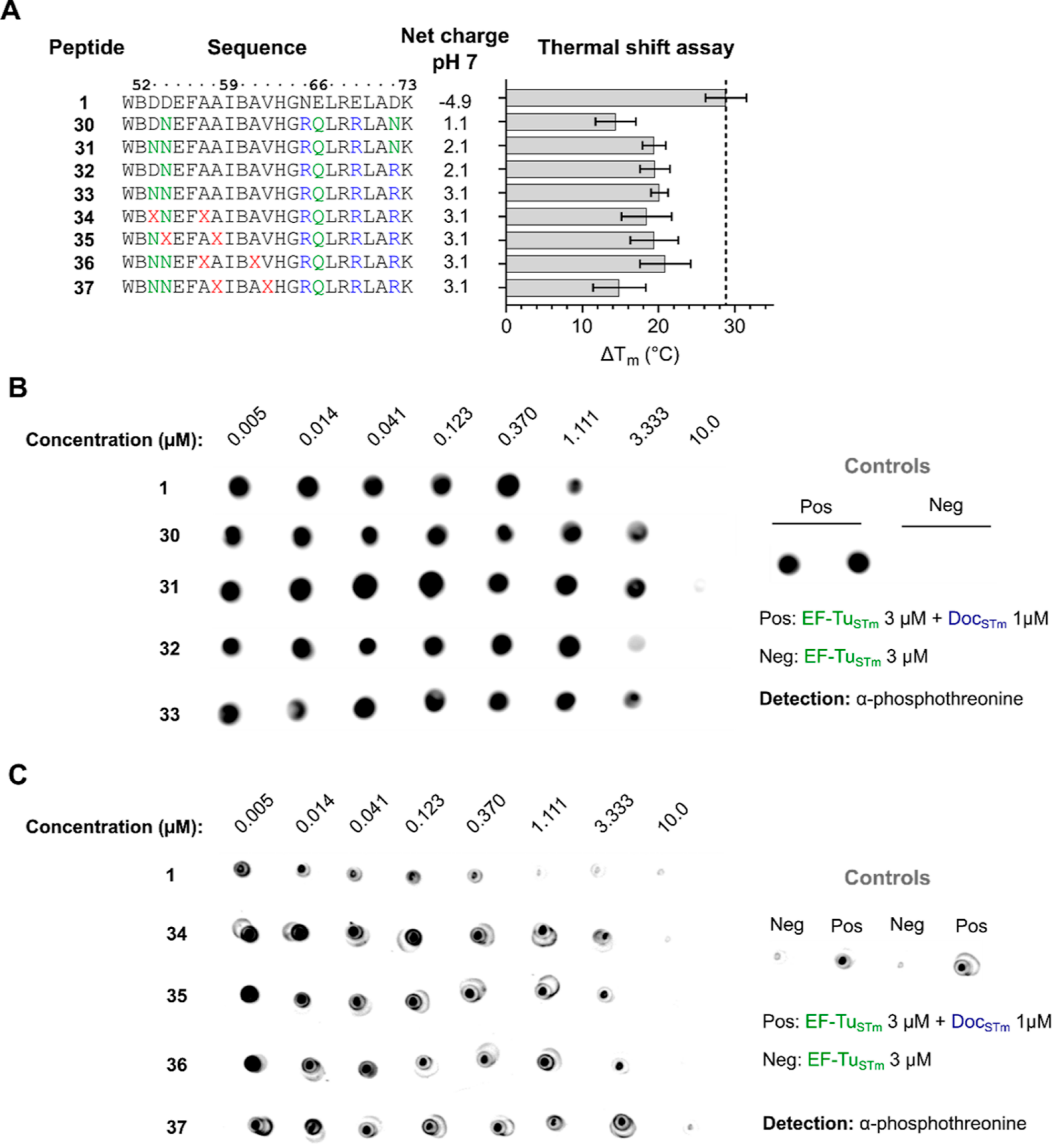
N-terminally hydrocarbon-stapled and C-terminally arginine-spiked Phd^52–73^ peptides with in vitro Doc_STm_ inhibitory activity. (A) Peptide sequences, peptide net charge at pH 7, and Δ*T*_m_ (thermal shift assay) values of the interaction of Doc_STm_ to Phd^52–73^ peptide 1 and analogues 30–37. Δ*T*_m_ values are shown as mean ± SD. B = l-norleucine, and X = (*S*)-2-(4-pentenyl)-alanine. (B) Dot blot detection of phosphorylated EF-Tu_STm_ in the presence of Doc_STm_ and Phd^52–73^ peptides 1 and 30–33. Peptides were tested at eight concentrations, ranging from 10 μM to 5 nM (3-fold dilutions). Negative (EF-Tu_STm_ 3 μM) and positive (EF-Tu_STm_ 3 μM + Doc_STm_ 1 μM) phosphorylation controls of the assay are shown on the right. (C) Dot blot detection of phosphorylated EF-Tu_STm_ in the presence of Doc_STm_ and Phd^52–73^ peptides 1 and 34–37. Assay controls are shown on the right.

Residues Asn65/Glu69 or Asn65/Glu69/Asp72 in peptide **1** were substituted for arginine and mixed with different combinations of substitutions of asparagine/glutamine equivalents of Asp53, Asp54, Glu66, and Asp72 to yield peptides **30**–**33** with net charges ranging from 1.1 to 3.1 (Figure 5A). The substitutions in **31**–**33** were well tolerated (Δ*T*_m_ ≈ 20 °C) similar to **26**–**29**, while **30** exhibited a reduced stabilization of Doc_STm_ (Δ*T*_m_ ≈ 14 °C). A concentration higher than three times the Doc_STm_ concentration was required for full Doc_STm_ inhibition by **30**, **31,** and **33** in the EF-Tu_STm_ phosphorylation assay, while **32** displayed a slightly higher Doc inhibition activity close to the activity of peptide **1** (Figure 5B).

Peptide **33** with good Doc_STm_ stabilization and the highest positive net charge was subsequently used for stapling evaluation: *i*, *i* + 4 hydrocarbon stapling was performed in all possible positions in the N-terminal half of the peptide up to the central glycine (Figure 5A), while keeping the hot-spot residues intact. The resulting stapled analogues **34**–**37** displayed a Doc inhibition activity similar to that of unstapled **33** (Figure 5C), i.e., reduced activity compared to peptide **1**. In addition, **34**–**36** stabilized Doc_STm_ to the same extent as **33** by thermal shift (Δ*T*_m_ ≈ 18–21 °C), while analogue **37** with the staple located closest to the suggested structural glycine-induced kink in the peptide displayed a reduced stabilization (Figure 5A).

FAM-labeled variants of the new peptide series **30**–**37** were tested for their cellular uptake into *E. coli*; however, precipitation was observed for all peptides in the assay conditions, resulting in large errors and an overestimation of the uptake (Figure S1).

### Cellular Uptake of Phd^52–73^ Peptide Analogues in *S. typhimurium* and Intracellular Doc_STm_ Inhibition Activity

**Figure 6:**
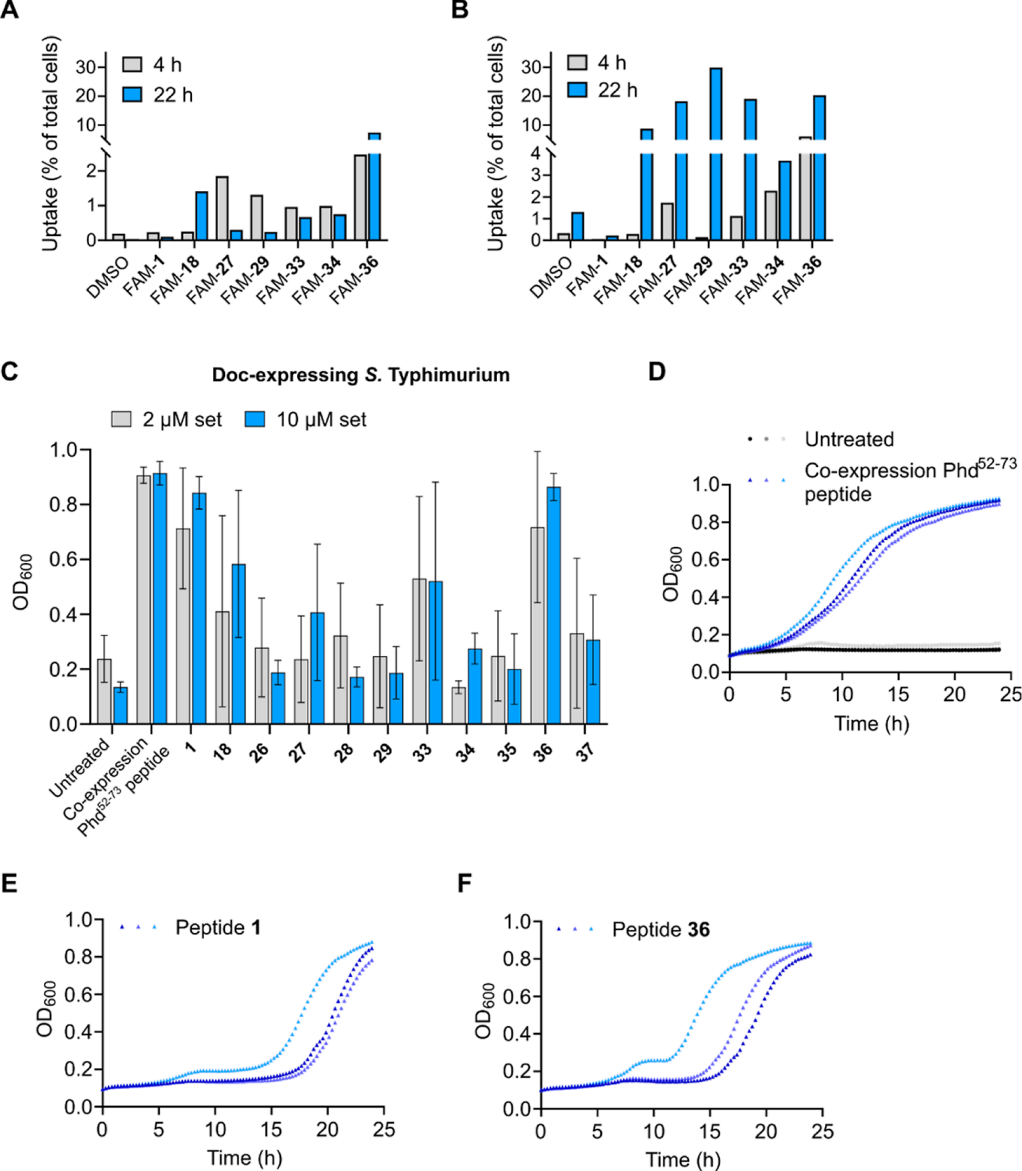
Cellular uptake and Doc_STm_inhibition activity of selected peptides in *S. typhimurium*. Cellular uptake of (A) 2 and (B) 10 μM selected fluorescein (FAM)-labeled Phd^52–73^ analogues in *S. typhimurium* after incubation for 4 or 22 h at RT as determined by flow cytometry. (C) Growth end points (measured as OD_600_) of a *S. typhimurium* (14028) Δphd-doc:Km strain expressing Doc_STm_ (pBAD33) after treatment with 2 or 10 μM selected peptides for 24 h at 37 °C. Untreated Doc_STm_-expressing *S. typhimurium* (negative control) as well as *S. typhimurium* (14028s) Δphd-doc:Km strain coexpressing Doc_STm_ and Phd_STm_^52–73^ antitoxin peptide (positive control) were included in each measurement. Data are shown as means ± SD (D) growth curves of untreated Doc_STm_-expressing *S. typhimurium* (black/gray) and *S. typhimurium* coexpressing Doc_STm_ and Phd_STm_^52–73^ peptide (blue) from three independent experiments. (E,F) Growth curves of Doc_STm_-expressing *S. typhimurium* were treated with 10 μM peptide **1** (E) or peptide **36** (F) from three independent experiments.

Selected Phd^52–73^ analogues with the highest Doc_STm_ binding affinities were investigated for their cellular uptake into Gram-negative target bacterium *S. typhimurium*. Bacteria were treated with fluorescein-labeled versions of peptide **1**, unstapled **33,** and stapled peptides **18**, **27**, **29**, **34,** and **36** at two different concentrations (2 and 10 μM). Uptake of peptides into the bacteria after 4 and 22 h, to determine both initial and longer-term uptake, was then measured by flow cytometry. As in the *E. coli* studies, highly negatively charged peptide **1** displayed very low uptake into *S. typhimurium* was present at both tested concentrations and incubation times. Additionally, at 2 μM, all modified peptides displayed very low to no uptake, except for stapled analogue **36**, which was taken up into around 7% of bacteria after 22 h of incubation (Figure 6A). At 10 μM concentration, peptides **18**, **27**, **29**, and **36** displayed higher uptake, with varying penetration efficiencies of around 9–30% (Figure 6B).

To investigate whether our Phd peptides could inhibit Doc toxin activity inside *S. typhimurium*, we performed growth rescue experiments. Peptide **1** and peptides **18** and **26**–**29** from the series of N-terminally arginine-spiked and C-terminally stapled Phd_STm_^52–37^ analogues, as well as peptides **33**–**37** from the series of N-terminally stapled and C-terminally arginine-spiked analogues, were tested in the growth rescue assay. A culture of Doc_STm_-expressing *S. typhimurium* strain was diluted to an OD_600_ of 0.1 and treated with 2 or 10 μM peptide for 24 h at 37 °C while growth of the bacteria was monitored via OD_600_. Untreated *S. typhimurium* displayed a clear growth arrest after 24 h of culture due to the activity of Doc_STm_ toxin (Figure 6C,D). Coexpression of a wild-type Phd_STm_^52–37^ peptide in *S. typhimurium* as a positive control for Doc inhibition resulted in growth rescue, as displayed by a final OD_600_ of around 0.9 after 24 h of culture (Figure 6C,D). Despite the low cell uptake, consistent and full rescue from Doc_STm_-induced growth inhibition was obtained for base peptide **1** after 24 h of treatment when used at 10 μM concentration (Figure 5C,E). The stapled peptide with the most promising uptake profile in *S. typhimurium*, **36**, reproducibly rescued Doc_STm_-induced growth arrest (Figure 6C,F). For all other peptides, we observed a larger variation and predominantly lower growth rescue activity (Figures S2 and S3). Analysis of the growth curves from independent experiments (Figure 6D–F) shows that growth rescue kinetics varied between experiments, with peptide **36** displaying faster growth rescue than peptide **1** in all experiments. Growth rescue by peptide **36** was slower than coexpression of a Phd_STm_^52–37^ peptide, which is expected due to the time required for bacterial cell penetration.

## Discussion

The generation of cell-permeable ligands for bacterial targets is a significant challenge, particularly for Gram-negative organisms that present a peptidoglycan layer sandwiched between an inner and an outer membrane as a barrier. For peptide ligands, the majority of effort has been focused on the development of antimicrobial peptides which destabilize these membranes to bring about a bactericidal effect.^33^ However, for many targets and applications, bacterial cell penetration without any associated toxicity would be highly desirable. One such class of targets is bacterial toxins, which are involved in stress responses. Peptide ligands have been developed which work to activate toxins with the aim of initiating cell death.^9,34,35^ However, we propose an alternative approach. Given that toxin activation may result in the increased formation of antibiotic tolerance persister cells,^36^ we have instead focused on the development of toxin inhibitors.

In this work, we sought to develop cell-penetrant peptide inhibitors of the bacterial toxin Doc, a target implicated in the survival of *Salmonella* to antibiotic treatment.^23^ Taking the Doc_STm_ binding sequence from its cognate antitoxin, Phd, as a starting point, we carried out a series of modifications with the aim of improving uptake into Gram-negative *Salmonella* while retaining the high affinity and inhibition activity of the wild type. As our initial sequence, **1** had a net charge of −4.9 at pH 7, and knowing how crucial positive charges are for cell uptake, we initially performed an arginine scan to determine the tolerance of the sequence for positive charge substitutions. With this and our previous work characterizing the interaction hot spot residues^31^ in hand, we then synthesized a library of peptides with two or three arginine substitutions in combination with the replacement of native carboxylic acid residues (Asp and Glu) for equivalent amide side chains (Asn and Gln) and hydrocarbon peptide stapling. In all cases, this resulted in varying degrees of reduction in Doc_STm_ stabilization and inhibition activity in vitro when compared to peptide **1**. Fortunately, in many cases these reductions were tolerated, yielding peptides capable of stabilizing Doc_STm_ with a melting temperature shift (Δ*T*_m_) of more than 20 °C and successfully inhibiting the toxin, despite possessing net charges of between +1.1 and +3.1 at pH 7 (**18**, **26**–**29**, **33**–**37**).

A subset of fluorescein-labeled analogues of these peptides showed promising improvements in uptake in *S. typhimurium* in comparison with peptide **1**, which displayed less than 1% fluorescent cells after 22 h at a concentration of 10 μM. In contrast, when treated with 2 μM of peptide **36**, approximately 10% of cells contained peptide after 22 h, and when treated with 10 μM of peptide **36**, more than 20% of cells showed evidence of internalized peptide (Figure 5A,B). We then treated *S. typhimurium* cells expressing Doc_STm_ with peptides **1**, **18**, **26**–**29**, **33**–**37**. In the absence of Doc_STm_ inhibition, bacterial growth and replication were halted, and OD_600_ remained static over the time course of the experiment. When Phd_STm_^52–73^ was coexpressed with Doc_STm_, growth was rescued, and the cultures reached an OD_600_ of ∼0.9 after 24 h (Figure 5D). When treated with either peptide **1** or **36**, growth rescue was also observed and the same maximal OD_600_ was reached after 24 h in both cases, albeit with a longer lag time (Figure 5E). Stapled peptide **36** was able to rescue growth more rapidly than **1**, indicating that the improved cell uptake enhances the in vivo activity (Figure 5F). Given the poor uptake of FAM-**1**, the observed Doc inhibition was somewhat surprising. It is possible that the hydrophobic fluorescein hinders bacterial cell penetration, resulting in an underestimation in peptide cell penetration, and/or that the higher affinity and activity of this sequence (closest to the native antitoxin) ensured that any which does permeate a bacterial cell is highly effective in binding to and inhibiting the toxin.

## Conclusions

We have developed a novel class of Phd_STm_ peptides that effectively inhibit, both in vitro and in vivo, the toxin Doc_STm_, which contributes to the antibiotic survival of *S. typhimurium*. By using a combination of amino acid substitutions and hydrocarbon stapling, we have significantly improved the uptake of the wild-type sequence to enable the extracellular administration of our Doc_STm_ inhibitors. The most effective peptide, **36**, fully rescued Doc_STm_-induced growth inhibition at a faster rate than the unmodified peptide **1**. This paves the way for the application of stapled peptides as toxin inhibitors to reduce the antibiotic tolerance of pathogenic bacteria.

## Materials and Methods

### Peptide Synthesis

#### Materials

9-Fluorenylmethoxycarbonyl (Fmoc)-protected amino acids were purchased from CEM (Buckingham, United Kingdom), Fluorochem (Hadfield, United Kingdom), and Sigma-Aldrich (Gillingham, United Kingdom), and preloaded Fmoc-Lys(Boc)-Wang resin was from Novabiochem (Watford, United Kingdom). *N*,*N*′-Diisopropylcarbodiimide (DIC) was from Fluorochem, piperidine, acetic anhydride, triisopropylsilane (TIS), formic acid, anhydrous 1,2-dichloroethane (DCE), Grubbs first-generation catalyst, and 5(6)-carboxyfluorescein (FAM) were from Sigma-Aldrich, and ethyl 2-cyano-2-(hydroxyimino)acetate (Oxyma Pure) was obtained from CEM. Acetonitrile (ACN), dimethylformamide (DMF), dimethyl sulfoxide (DMSO), and diethyl ether were from VWR (Lutterworth, United Kingdom), and dichloromethane (DCM) and trifluoroacetic acid (TFA) were obtained from Fisher Scientific UK (Loughborough, United Kingdom).

#### Solid-Phase Synthesis, Purification, and Analysis of Peptides

Peptides were synthesized by solid-phase peptide synthesis (SPPS) using a Liberty Blue automated microwave peptide synthesizer (CEM) and the standard 9-fluorenylmethoxycarbonyl/*tert*-Butyl (Fmoc/*t*Bu) strategy. Fmoc-Lys(Boc)-Wang resin (50 μmol scale, 0.64 mmol/g) was used to obtain peptides as C-terminal acids. In general, a 5-fold molar excess of Fmoc-amino acid (250 μmol, 0.2 M solution in DMF) was coupled with 5 eq. Oxyma Pure (250 μmol, 0.5 M solution in DMF) and 10 eq. DIC (500 μmol, 0.5 M solution in DMF) in DMF for 2 min (single coupling) or 2 × 2 min (double coupling) at 90 °C. Fmoc-(*S*)-2-(4-pentenyl)Ala-OH was coupled for 5 min (single coupling) at 90 °C, with the subsequent amino acid being coupled for 2 × 5 min (double coupling) at 90 °C.

N-terminal Fmoc deprotection was accomplished by using 10% (*v*/*v*) piperidine, 0.1 M Oxyma Pure in DMF for 60–90 s at 90 °C. Following automated SPPS, peptides were manually acetylated at the N-terminus with 5% acetic anhydride in DMF for 3 × 15 min. For the generation of fluorescein-labeled peptides, 25 μmol of N-terminally deprotected peptide resins were used and 3 equiv 5(6)-carboxyfluorescein (FAM, 75 μmol) were manually coupled to the N-terminus with 5 equiv DIC (125 μmol) and 5 equiv Oxyma Pure (125 μmol) in DMF overnight at RT.

To generate hydrocarbon-stapled peptides, N-terminally Fmoc-protected resin-bound peptides (50 μmol scale) with (*S*)-2-(4-pentenyl)-alanine residues in positions *i*, *i* + 4 were washed with DCM and anhydrous DCE. Ring-closing metathesis was then performed by treatment of the peptide resins with 10 mM Grubbs first-generation catalyst in anhydrous DCE (1 mL) for 2 × 2–3 h at RT under nitrogen atmosphere. Following the reaction, peptide resins were extensively washed with DCE and DCM, the N-terminal Fmoc group was removed, and peptides were either acetylated or coupled to 5(6)-carboxyfluorescein as described above.

Cleavage from the resin and simultaneous side chain deprotection were accomplished using a mixture of TFA/TIS/H_2_O (95/2.5/2.5, 3 mL) for 45 min at 40 °C in the CEM Razor rapid peptide cleavage system or for 3 h at RT. The cleavage mixture was concentrated to around 1 mL under a nitrogen stream, and crude peptides were precipitated and washed with ice-cold diethyl ether. The peptide pellets were dissolved in ACN/H_2_O and subsequently lyophilized. Crude peptides were purified on a Shimadzu LC-20AR preparative HPLC system using a preparative reversed-phase Phenomenex Aeris Peptide XB-C18 column (150 × 21 mm, 5 μm, 100 Å) with a flow rate of 20 mL/min, different linear gradients of eluent B1 [0.08% (*v*/*v*) TFA in ACN] in eluent A1 [0.1% (*v*/*v*) TFA in water], and detection at 220 nm. The purity of the peptides was determined on a Shimadzu LC-2030C 3D HPLC system using an analytical Phenomenex Aeris Peptide XB-C18 column (150 × 4.6 mm, 3.6 μM, 100 Å) with a flow rate of 1.5 mL/min, a linear gradient of 20 to 95% eluent B1 in eluent A1 over 15 min, and detection at 220 nm. The correct identity of the peptides was confirmed on a Waters LC–MS system (2545 quaternary gradient module, 2767 sample manager, system fluidics organizer, and 3100 mass detector) using an analytical reversed-phase Waters XBridge C18 column (100 × 4.6 mm, 5 μm, 130 Å, 1.2 mL/min), a linear gradient of 20 to 98% eluent B2 [0.1% (*v*/*v*) formic acid in ACN] in eluent A2 [0.1% (*v*/*v*) formic acid in water] over 10 min, and mass detection in the range from 400 to 2000 *m*/*z*, as well as by MALDI-ToF mass spectrometry (Micromass, Waters). The observed masses were in agreement with the calculated masses, and a purity of >93% could be obtained for all compounds by LC–MS analysis (Tables S1 and S2).

#### Protein Expression and Purification

C-terminally His-tagged *Salmonella* Typhimurium Doc toxin and EF-Tu protein were recombinantly produced in *E. coli* as previously described.^31^

### Biochemical and Biological Methods

#### Thermal Shift Assay

The thermal shift assay was performed using a Mx3005P qPCR System (Agilent) collecting fluorescence data with a temperature ramp of 25 to 95 °C. Samples were prepared in a buffer containing 20 mM K_2_HPO_4_ and 50 mM (NH_4_)_2_SO_4_ at pH 8.0, with a final concentration of recombinant Doc protein at 5 μM and Phd peptides at 50 μM. SYPRO Orange dye (Sigma-Aldrich, 5000X stock in DMSO) was used to monitor protein denaturation in a final concentration of 3×. The final sample volume was 20 μL. Each condition was prepared in triplicate in each independent experiment. The melting curves were plotted using GraphPad Prism 9 (GraphPad Software, USA), and the melting temperatures were obtained by fitting the sigmoidal section of the curves to a Boltzmann sigmoid function. For the calculation of thermal shifts Δ*T*_m_, the average melting temperature of free Doc_STm_ toxin as determined in the respective peptide measurement cycles was subtracted from the average melting temperature of Doc_STm_ in the presence of the peptide (Table S3).

#### Dot Blot Phosphorylation Assay

Samples were prepared in assay buffer [50 mM HEPES (pH 7.5), 25 mM (NH_4_)_2_SO_4_, 2 mM TCEP, 2 mM MgCl_2_, and 1 mM ATP] with a final concentration of recombinant Doc at 1 μM, recombinant EF-Tu at 3 μM, and varying concentrations of synthetic Phd peptides (final concentrations: 10 μM to 5 nM). EF-Tu (3 μM) in assay buffer was used as a negative control (no Doc and Phd peptide), while EF-Tu (3 μM) with Doc (1 μM) in assay buffer was used as a positive control (no Doc inhibitor). The final sample volume was 10 μL. Samples were incubated for 16 h at RT and subsequently spotted on a nitrocellulose membrane. Phosphorylated EF-Tu was detected by immunodecoration using a rabbit monoclonal antiphosphothreonine antibody (Abcam, ab218195) at 1:2000 dilution (1 h at 4 °C), followed by incubation with a goat antirabbit IgG (H + L) HRP conjugate antibody (Advansta) at 1:10,000 dilution (1 h at RT). Chemiluminescence was developed using the HRP Luminata Kit (Merck, WBLUR0100) and captured with an ImageQuant LAS4000 Western blot imaging system (GE HealthCare). Each peptide was tested in two independent experiments.

#### Peptide Uptake in *E. coli*

*E. coli* MG1655 strain was grown in LB medium to an OD_600_ of around 0.5. Approximately 10^7^ cells were washed once with PBS and subsequently incubated with 5 μM of the indicated 5(6)-carboxyfluorescein-labeled Phd^52–73^ peptide in PBS (500 μL) for 2 h at 37 °C. Bacteria were washed twice with PBS, and trypan blue (1 mg mL^–1^) in PBS was added and incubated for 10 min at RT. Bacteria were washed an additional time with PBS and resuspended in 2 mL of PBS. Flow cytometry analysis was performed using a ThermoFisher Attune NxT and a blue laser (BL1) for fluorescein excitation.

#### Peptide Uptake in *S. typhimurium*

*S. typhimurium* (14028s) *glmS::mCherry* strain was grown overnight in LB medium containing 1% glucose. The culture was then diluted to an OD_600_ of approximately 0.1 into 200 μL of fresh M9 minimal medium supplemented with 0.5% arabinose containing 2 or 10 μM of the indicated 5(6)-carboxyfluorescein-labeled Phd^52–73^ peptide. The samples were incubated on the bench at RT for 4 or 22 h with no access to light. Bacteria were then spun down, washed with 1 mL of PBS solution, and finally resuspended in 1 mL of PBS solution for flow cytometry analysis (BD LSR II). Constitutively expressed mCherry was used to discriminate bacteria from the debris in each sample.

#### Growth Rescue Experiments in *S. typhimurium*

*S. typhimurium* (14028s) Δ*phd-doc::Km* strains carrying pCA24N (empty vector or encoding for the Phd^52–73^ variant) and pBAD33::doc plasmids were grown overnight in LB medium containing 1% glucose and supplemented with 100 μg/mL carbenicillin and 34 μg/mL chloramphenicol antibiotics. Cultures were then diluted to an OD_600_ of approximately 0.1 into 200 μL of fresh M9 minimal medium supplemented with 0.5% arabinose, 100 μg/mL carbenicillin, 34 μg/mL chloramphenicol, and containing 2 or 10 μM of the indicated synthetic Phd^52–73^ peptide. Untreated Doc_STm_-expressing *S. typhimurium* as well as *S. typhimurium* coexpressing Doc and Phd^52–73^ peptides were included as controls in each experiment. After 2 h of incubation at RT, samples were transferred to a flat-bottom 96-well plate (Greiner), and OD_600_ was monitored every 15 min for 24 h at 37 °C using an Infinite M Plex plate reader (Tecan LifeScience) and orbital shaking with 1 mm amplitude. Peptides were tested in three independent experiments.

## Supporting Information Available

Peptide update in *E. coli*, analytical characterization of the synthetic Phd peptides, detailed DSF data, additional growth rescue experiment data, replicate EF-Tu phosphorylation assays, and links to online data repository (PDF)

## References

[ref1] Azzarito V.; Long K.; Murphy N. S.; Wilson A. J. Inhibition of Alpha-Helix-Mediated Protein-Protein Interactions Using Designed Molecules. Nat. Chem. 2013, 5 (3), 161–173. 10.1038/nchem.1568. 23422557

[ref2] Skwarczynska M.; Ottmann C. Protein-Protein Interactions as Drug Targets. Future Med. Chem. 2015, 7 (16), 2195–2219. 10.4155/fmc.15.138. 26510391

[ref3] Ali A. M.; Atmaj J.; Van Oosterwijk N.; Groves M. R.; Dömling A. Stapled Peptides Inhibitors: A New Window for Target Drug Discovery. Comput. Struct. Biotechnol. J. 2019, 17, 263–281. 10.1016/j.csbj.2019.01.012. 30867891 PMC6396041

[ref4] Bluntzer M. T. J.; O’Connell J.; Baker T. S.; Michel J.; Hulme A. N. Designing Stapled Peptides to Inhibitprotein-Proteininteractions: An Analysis of Successes in a Rapidly Changing Field. Pept. Sci. 2021, 113 (1), e24191 10.1002/pep2.24191.

[ref5] Luo X.; Chen H.; Song Y.; Qin Z.; Xu L.; He N.; Tan Y.; Dessie W. Advancements, Challenges and Future Perspectives on Peptide-Based Drugs: Focus on Antimicrobial Peptides. Eur. J. Pharm. Sci. 2023, 181, 106363 10.1016/j.ejps.2022.106363. 36529161

[ref6] Moiola M.; Memeo M. G.; Quadrelli P. Stapled Peptides—A Useful Improvement for Peptide-Based Drugs. Molecules 2019, 24 (20), 3654 10.3390/molecules24203654. 31658723 PMC6832507

[ref7] Bird G. H.; Mazzola E.; Opoku-Nsiah K.; Lammert M. A.; Godes M.; Neuberg D. S.; Walensky L. D. Biophysical Determinants for Cellular Uptake of Hydrocarbon-Stapled Peptide Helices. Nat. Chem. Biol. 2016, 12 (10), 845–852. 10.1038/nchembio.2153. 27547919 PMC5055751

[ref8] Payne S. R.; Pau D. I.; Whiting A. L.; Kim Y. J.; Pharoah B. M.; Moi C.; Boddy C. N.; Bernal F. Inhibition of Bacterial Gene Transcription with an RpoN-Based Stapled Peptide. Cell Chem. Biol. 2018, 25 (9), 1059–1066.e4. 10.1016/j.chembiol.2018.05.007. 29887265 PMC6151150

[ref9] Kang S.-M.; Moon H.; Han S.-W.; Kim D.-H.; Kim B. M.; Lee B.-J. Structure-Based De Novo Design of Mycobacterium Tuberculosis VapC-Activating Stapled Peptides. ACS Chem. Biol. 2020, 15 (9), 2493–2498. 10.1021/acschembio.0c00492. 32840352

[ref10] Robertson N. S.; Spring D. R. Using Peptidomimetics and Constrained Peptides as Valuable Tools for Inhibiting Protein-Protein Interactions. Molecules 2018, 23 (4), 959 10.3390/molecules23040959. 29671834 PMC6017787

[ref11] Wang H.; Dawber R. S.; Zhang P.; Walko M.; Wilson A. J.; Wang X. Peptide-Based Inhibitors of Protein-Protein Interactions: Biophysical, Structural and Cellular Consequences of Introducing a Constraint. Chem. Sci. 2021, 12 (17), 5977–5993. 10.1039/D1SC00165E. 33995995 PMC8098664

[ref12] Payton M.; Pinchasik D.; Mehta A.; Goel S.; Zain J. M.; Sokol L.; Jacobsen E.; Patel M. R.; Horwitz S. M.; Meric-Bernstam F.; Shustov A.; Weinstock D.; Aivado M.; Annis D. A. Phase 2a study of a novel stapled peptide ALRN-6924 disrupting MDMX- and MDM2-mediated inhibition of wild-type TP53 in patients with peripheral t-cell lymphoma. Ann. Oncol. 2017, 28 (suppl_5), v370 10.1093/annonc/mdx373.045.

[ref13] Saleh M. N.; Patel M. R.; Bauer T. M.; Goel S.; Falchook G. S.; Shapiro G. I.; Chung K. Y.; Infante J. R.; Conry R. M.; Rabinowits G.; Hong D. S.; Wang J. S.; Steidl U.; Walensky L. D.; Naik G.; Guerlavais V.; Vukovic V.; Annis D. A.; Aivado M.; Meric-Bernstam F. Phase 1 Trial of ALRN-6924, a Dual Inhibitor of MDMX and MDM2, in Patients with Solid Tumors and Lymphomas Bearing Wild-Type TP53. Clin. Cancer Res. 2021, 27 (19), 5236–5247. 10.1158/1078-0432.CCR-21-0715. 34301750 PMC9401461

[ref14] Douse C. H.; Maas S. J.; Thomas J. C.; Garnett J. A.; Sun Y.; Cota E.; Tate E. W. Crystal Structures of Stapled and Hydrogen Bond Surrogate Peptides Targeting a Fully Buried Protein-Helix Interaction. ACS Chem. Biol. 2014, 9 (10), 2204–2209. 10.1021/cb500271c. 25084543

[ref15] Ovchinnikov V.; Stone T. A.; Deber C. M.; Karplus M. Structure of the EmrE Multidrug Transporter and Its Use for Inhibitor Peptide Design. Proc. Natl. Acad. Sci. U.S.A. 2018, 115 (34), E7932–E7941. 10.1073/pnas.1802177115. 30082384 PMC6112734

[ref16] Paulussen F. M.; Schouten G. K.; Moertl C.; Verheul J.; Hoekstra I.; Koningstein G. M.; Hutchins G. H.; Alkir A.; Luirink R. A.; Geerke D. P.; van Ulsen P.; den Blaauwen T.; Luirink J.; Grossmann T. N. Covalent Proteomimetic Inhibitor of the Bacterial FtsQB Divisome Complex. J. Am. Chem. Soc. 2022, 144 (33), 15303–15313. 10.1021/jacs.2c06304. 35945166 PMC9413201

[ref17] Mourtada R.; Herce H. D.; Yin D. J.; Moroco J. A.; Wales T. E.; Engen J. R.; Walensky L. D. Design of Stapled Antimicrobial Peptides That Are Stable, Nontoxic and Kill Antibiotic-Resistant Bacteria in Mice. Nat. Biotechnol. 2019, 37 (10), 1186–1197. 10.1038/s41587-019-0222-z. 31427820 PMC7437984

[ref18] Wojciechowska M.; Macyszyn J.; Miszkiewicz J.; Grzela R.; Trylska J. Stapled Anoplin as an Antibacterial Agent. Front. Microbiol. 2021, 12, 772038 10.3389/fmicb.2021.772038. 34966367 PMC8710804

[ref19] Hirano M.; Saito C.; Yokoo H.; Goto C.; Kawano R.; Misawa T.; Demizu Y. Development of Antimicrobial Stapled Peptides Based on Magainin 2 Sequence. Molecules 2021, 26 (2), 444 10.3390/molecules26020444. 33466998 PMC7830303

[ref20] Hu Y.; Li H.; Qu R.; He T.; Tang X.; Chen W.; Li L.; Bai H.; Li C.; Wang W.; Fu G.; Luo G.; Xia X.; Zhang J. Lysine Stapling Screening Provides Stable and Low Toxic Cationic Antimicrobial Peptides Combating Multidrug-Resistant Bacteria In Vitro and In Vivo. J. Med. Chem. 2022, 65 (1), 579–591. 10.1021/acs.jmedchem.1c01754. 34968054

[ref21] Schouten G. K.; Paulussen F. M.; Kuipers O. P.; Bitter W.; Grossmann T. N.; van Ulsen P. Stapling of Peptides Potentiates the Antibiotic Treatment of Acinetobacter Baumannii In Vivo. Antibiotics 2022, 11 (2), 273 10.3390/antibiotics11020273. 35203875 PMC8868297

[ref22] Fraikin N.; Goormaghtigh F.; Van Melderen L. Type II Toxin-Antitoxin Systems: Evolution and Revolutions. J. Bacteriol. 2020, 202( (7), ). 10.1128/JB.00763-19. PMC7167474 31932311

[ref23] Helaine S.; Cheverton A. M.; Watson K. G.; Faure L. M.; Matthews S. A.; Holden D. W. Internalization of Salmonella by Macrophages Induces Formation of Nonreplicating Persisters. Science 2014, 343 (6167), 204–208. 10.1126/science.1244705. 24408438 PMC6485627

[ref24] Ronneau S.; Helaine S. Clarifying the Link between Toxin-Antitoxin Modules and Bacterial Persistence. J. Mol. Biol. 2019, 431 (18), 3462–3471. 10.1016/j.jmb.2019.03.019. 30914294

[ref25] Lobato-Márquez D.; Díaz-Orejas R.; García-del Portillo F. Toxin-Antitoxins and Bacterial Virulence. FEMS Microbiol. Rev. 2016, 40 (5), 592–609. 10.1093/femsre/fuw022. 27476076

[ref26] Page R.; Peti W. Toxin-Antitoxin Systems in Bacterial Growth Arrest and Persistence. Nat. Chem. Biol. 2016, 12 (4), 208–214. 10.1038/nchembio.2044. 26991085

[ref27] Li T.; Yin N.; Liu H.; Pei J.; Lai L. Novel Inhibitors of Toxin HipA Reduce Multidrug Tolerant Persisters. ACS Med. Chem. Lett. 2016, 7 (5), 449–453. 10.1021/acsmedchemlett.5b00420. 27190591 PMC4867474

[ref28] Równicki M.; Lasek R.; Trylska J.; Bartosik D. Targeting Type II Toxin-Antitoxin Systems as Antibacterial Strategies. Toxins 2020, 12 (9), 568 10.3390/toxins12090568. 32899634 PMC7551001

[ref29] Garcia-Pino A.; Christensen-Dalsgaard M.; Wyns L.; Yarmolinsky M.; Magnuson R. D.; Gerdes K.; Loris R. Doc of Prophage P1 Is Inhibited by Its Antitoxin Partner Phd through Fold Complementation. J. Biol. Chem. 2008, 283 (45), 30821–30827. 10.1074/jbc.M805654200. 18757857 PMC2576525

[ref30] Castro-Roa D.; Garcia-Pino A.; De Gieter S.; van Nuland N. A. J.; Loris R.; Zenkin N. The Fic Protein Doc Uses an Inverted Substrate to Phosphorylate and Inactivate EF-Tu. Nat. Chem. Biol. 2013, 9 (12), 811–817. 10.1038/nchembio.1364. 24141193 PMC3836179

[ref31] de Castro G. V.; Worm D. J.; Grabe G. J.; Rowan F. C.; Haggerty L.; de la Lastra A. L.; Popescu O.; Helaine S.; Barnard A. Characterization of the Key Determinants of Phd Antitoxin Mediated Doc Toxin Inactivation in Salmonella. ACS Chem. Biol. 2022, 17 (6), 1598–1606. 10.1021/acschembio.2c00276. 35647667 PMC9207808

[ref32] Walensky L. D.; Bird G. H. Hydrocarbon-Stapled Peptides: Principles, Practice, and Progress. J. Med. Chem. 2014, 57 (15), 6275–6288. 10.1021/jm4011675. 24601557 PMC4136684

[ref33] Lee H.-M.; Ren J.; Tran K. M.; Jeon B.-M.; Park W.-U.; Kim H.; Lee K. E.; Oh Y.; Choi M.; Kim D.-S.; Na D. Identification of Efficient Prokaryotic Cell-Penetrating Peptides with Applications in Bacterial Biotechnology. Commun. Biol. 2021, 4 (1), 205–213. 10.1038/s42003-021-01726-w. 33589718 PMC7884711

[ref34] Kang S.-M.; Moon H.; Han S.-W.; Kim B. W.; Kim D.-H.; Kim B. M.; Lee B.-J. Toxin-Activating Stapled Peptides Discovered by Structural Analysis Were Identified as New Therapeutic Candidates That Trigger Antibacterial Activity against Mycobacterium Tuberculosis in the Mycobacterium Smegmatis Model. Microorganisms 2021, 9 (3), 568 10.3390/microorganisms9030568. 33801872 PMC8000039

[ref35] Kang S.-M.; Jin C.; Kim D.-H.; Park S. J.; Han S.-W.; Lee B.-J. Structure-Based Design of Peptides That Trigger Streptococcus Pneumoniae Cell Death. FEBS J. 2021, 288 (5), 1546–1564. 10.1111/febs.15514. 32770723 PMC7984235

[ref36] Ronneau S.; Helaine S. Clarifying the Link between Toxin-Antitoxin Modules and Bacterial Persistence. J. Mol. Biol. 2019, 431, 3462–3471. 10.1016/j.jmb.2019.03.019. 30914294

