# Supplementary material for: Stapled Phd Peptides Inhibit Doc Toxin Induced Growth Arrest in Salmonella
Source: ACS Chem Biol. 2023 Nov 21;18(12):2485–94. doi: 10.1021/acschembio.3c00411 (PMC10728895; doi:10.1021/acschembio.3c00411)
Supplement: Supplementary file 1 — cb3c00411_si_001.pdf [file cb3c00411_si_001.pdf]

Supporting Information

Stapled Phd peptides inhibit Doc toxin induced growth arrest in *Salmonella*

Dennis J. Worm[1], Grzegorz J. Grabe[2], Guilherme Vieira de Castro[1], Sofya Rabinovich[1], Ian Warm[1], Kira Isherwood[1], Sophie Helaine[2] and Anna Barnard[1]*

1) Department of Chemistry, Molecular Sciences Research Hub, Imperial College London, 82 Wood Lane, London W12 0BZ, United Kingdom

2) Department of Microbiology, Harvard Medical School, 4 Blackfan Circle, Boston, MA 02115, USA

Address for Correspondence: a.barnard@imperial.ac.uk

Contents

1. Supplementary figures 2
2. Peptide characterisation 3
3. Thermal shift assay 7
4. Salmonella growth rescue experiment 9
5. EF-Tu phosphorylation assay 11
6. Online data repository 13

1. Supplementary figures

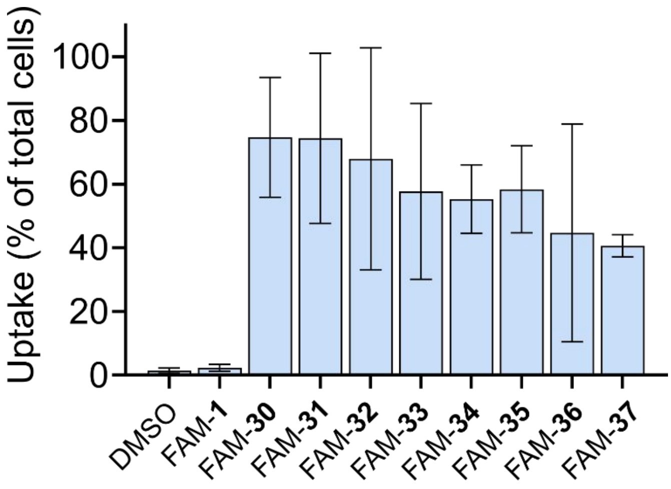

Figure S1. Bacterial uptake of Phd$^{52\text{-}73}$ analogues in *E. coli*. Cellular uptake of 5 µM carboxyfluorescein (FAM)-labelled Phd$^{52\text{-}73}$ peptides **30-37** in *E. coli* MG1655 after incubation for 2 hours at 37°C as determined by flow cytometry. Data is shown as means ± SD. Precipitation was observed for all peptides in the assay.

2. Peptide characterisation

Table S1. Analysis of pure Phd$^{52\text{-}73}$ peptides by analytical HPLC. A reversed-phase Phenomenex Aeris Peptide XB-C18 column (150 mm × 4.6 mm, 3.6 µM, 100 Å) with a flow rate of 1.5 mL/min and a linear gradient of 20% to 95% eluent B1 (0.08 % (*v/v*) TFA in ACN) in eluent A1 (0.1 % (*v/v*) TFA in water) over 15 min was used.

**Peptide**	**Sequence**	**t_R [min]**	**Purity**
**1**	Ac-WBDDEFAAIBAVHGNELRELADK-OH	5.74	97%
**FAM-1**	FAM-WBDDEFAAIBAVHGNELRELADK-OH	6.27	95%
**2**	Ac-WBRDEFAAIBAVHGNELRELADK-OH	5.50	99%
**3**	Ac-WBDREFAAIBAVHGNELRELADK-OH	5.53	99%
**4**	Ac-WBDDRFAAIBAVHGNELRELADK-OH	5.04	99%
**5**	Ac-WBDDEFRAIBAVHGNELRELADK-OH	5.36	99%
**6**	Ac-WBDDEFARIBAVHGNELRELADK-OH	5.38	99%
**7**	Ac-WBDDEFAAIBRVHGNELRELADK-OH	5.40	97%
**8**	Ac-WBDDEFAAIBARHGNELRELADK-OH	5.23	99%
**9**	Ac-WBDDEFAAIBAVHGRELRELADK-OH	5.57	99%
**10**	Ac-WBDDEFAAIBAVHGNRLRELADK-OH	5.33	99%
**11**	Ac-WBDDEFAAIBAVHGNELRRLADK-OH	5.50	99%
**12**	Ac-WBDDEFAAIBAVHGNELRELRDK-OH	5.33	99%
**13**	Ac-WBDDEFAAIBAVHGNELRELARK-OH	5.55	99%
**14**	Ac-WBRREFRRIBRVHGNELRELADK-OH	6.46	98%
**15**	Ac-WBRREFRRIBRVHGRELRELADK-OH	6.40	99%
**16**	Ac-WBRDEFAAIBRVHGNELRELADK-OH	5.31	98%
**FAM-16**	FAM-WBRDEFAAIBRVHGNELRELADK-OH	5.43	95%
**17**	Ac-WBRXEFAXIBRVHGNELRELADK-OH	5.72	95%
**FAM-17**	FAM-WBRXEFAXIBRVHGNELRELADK-OH	6.24	95%
**18**	Ac-WBRDEFAAIBRVHGXELRXLADK-OH	5.74	93%
**FAM-18**	FAM-WBRDEFAAIBRVHGXELRXLADK-OH	6.14	93%
**19**	Ac-WBRDEFRAIBRVHGNELRELADK-OH	4.86	99%
**FAM-19**	FAM-WBRDEFRAIBRVHGNELRELADK-OH	5.42	97%
**20**	Ac-WBRXEFRXIBRVHGNELRELADK-OH	5.50	99%
**FAM-20**	FAM-WBRXEFRXIBRVHGNELRELADK-OH	5.70	94%
**21**	Ac-WBRDEFRAIBRVHGXELRXLADK-OH	5.40	99%

Table S1 continued:

Peptide	Sequence	t_R [min]	Purity
**FAM-21**	FAM-WBRDEFRAIBRVHGXELRXLADK-OH	5.56	94%
**22**	Ac-WBRNQFAAIBRVHGNQLRELANK-OH	4.65	99%
**FAM-22**	FAM-WBRNQFAAIBRVHGNQLRELANK-OH	5.31	94%
**23**	Ac-WBRNQFAAIBRVHGXQLRXLANK-OH	5.14	99%
**FAM-23**	FAM-WBRNQFAAIBRVHGXQLRXLANK-OH	5.74	96%
**24**	Ac-WBRNQFRAIBRVHGNQLRELANK-OH	4.30	99%
**FAM-24**	FAM-WBRNQFRAIBRVHGNQLRELANK-OH	4.93	99%
**25**	Ac-WBRNQFRAIBRVHGXQLRXLANK-OH	4.84	99%
**FAM-25**	FAM-WBRNQFRAIBRVHGXQLRXLANK-OH	5.44	96%
**26**	Ac-WBRNEFAAIBRVHGNQLRELANK-OH	4.92	98%
**27**	Ac-WBRNEFAAIBRVHGXQLRXLANK-OH	5.44	98%
**28**	Ac-WBRNEFRAIBRVHGNQLRELANK-OH	4.59	98%
**29**	Ac-WBRNEFRAIBRVHGXQLRXLANK-OH	5.18	98%
**30**	Ac-WBDNEFAAIBAVHGRQLRRLANK-OH	5.00	97%
**FAM-30**	FAM-WBDNEFAAIBAVHGRQLRRLANK-OH	5.53	96%
**31**	Ac-WBNNEFAAIBAVHGRQLRRLANK-OH	4.81	98%
**FAM-31**	FAM-WBNNEFAAIBAVHGRQLRRLANK-OH	5.20	97%
**32**	Ac-WBDNEFAAIBAVHGRQLRRLARK-OH	4.85	98%
**FAM-32**	FAM-WBDNEFAAIBAVHGRQLRRLARK-OH	5.22	97%
**33**	Ac-WBNNEFAAIBAVHGRQLRRLARK-OH	4.73	97%
**FAM-33**	FAM-WBNNEFAAIBAVHGRQLRRLARK-OH	5.09	98%
**34**	Ac-WBXNEFXAIBAVHGRQLRRLARK-OH	7.22	95%
**FAM-34**	FAM-WBXNEFXAIBAVHGRQLRRLARK-OH	7.47	97%
**35**	Ac-WBNXEFAXIBAVHGRQLRRLARK-OH	5.82	94%
**FAM-35**	FAM-WBNXEFAXIBAVHGRQLRRLARK-OH	6.42	95%
**36**	Ac-WBNNEFXAIBXVHGRQLRRLARK-OH	5.51	96%
**FAM-36**	FAM-WBNNEFXAIBXVHGRQLRRLARK-OH	5.95	95%
**37**	Ac-WBNNEFAXIBAXHGRQLRRLARK-OH	4.93	98%
**FAM-37**	FAM-WBNNEFAXIBAXHGRQLRRLARK-OH	5.56	95%

B: L-norleucine, FAM: 5(6)-carboxyfluorescein, X: (*S*)-2-(4-pentenyl)alanine, t_R: Retention time.

Table S2. Analysis of pure Phd$^{52-73}$ peptides by LC-MS (ESI$^+$) and MALDI-ToF-MS.

Compound	M_{exact} (calc) [Da]	M_W (calc) [Da]	ESI$^+$ (LC-MS)	MALDI-ToF
			m/z (exp)	M_{exact} (exp) $[M+H]^+$
**1**	2666.3	2668.0	1335.0 (m/2), 890.5 (m/3)	2667.4
**FAM-1**	2982.4	2984.2	1492.7 (m/2), 995.5 (m/3)	2983.4
**2**	2707.4	2709.1	904.2 (m/3), 678.3 (m/4)	2708.5
**3**	2707.4	2709.1	1355.5 (m/2), 904.2 (m/3), 678.3 (m/4)	2708.6
**4**	2693.4	2695.0	1348.3 (m/2), 899.2 (m/3), 674.8 (m/4)	2694.8
**5**	2751.4	2753.1	1377.4 (m/2), 918.7 (m/3), 689.2 (m/4)	2752.8
**6**	2751.4	2753.1	1377.5 (m/2), 918.9 (m/3), 689.3 (m/4)	2753.1
**7**	2751.4	2753.1	1377.3 (m/2), 918.7 (m/3), 689.3 (m/4)	2752.8
**8**	2723.4	2725.0	1363.4 (m/2), 909.4 (m/3), 682.2 (m/4)	2724.9
**9**	2708.4	2710.1	1355.9 (m/2), 904.5 (m/3)	2709.05
**10**	2693.4	2695.0	1348.6 (m/2), 899.5 (m/3), 674.9 (m/4)	2694.6
**11**	2693.4	2695.0	1348.2 (m/2), 899.5 (m/3)	2694.6
**12**	2751.4	2753.1	1377.6 (m/2), 918.9 (m/3)	2753
**13**	2707.4	2709.1	1355.5 (m/2), 903.9 (m/3)	2708.6
**14**	3003.7	3005.5	1003.0 (m/3), 752.4 (m/4), 602.2 (m/5)	3004.7
**15**	3045.7	3047.6	1017.0 (m/3), 762.9 (m/4), 610.6 (m/5)	3046.8
**16**	2792.5	2794.2	932.5 (m/3), 699.8 (m/4), 560.1 (m/5)	2793.8
**FAM-16**	3108.5	3110.4	1038.2 (m/3), 778.9 (m/4), 623.4 (m/5)	3109.6
**17**	2856.6	2858.4	954.0 (m/3), 715.8 (m/4), 572.9 (m/5)	2857.6
**FAM-17**	3172.6	3174.6	1059.6 (m/3), 795.0 (m/4), 636.0 (m/5)	3174.6
**18**	2799.6	2801.3	n.d.	2800.4
**FAM-18**	3115.6	3117.6	1040.5 (m/3), 780.7 (m/4), 624.7 (m/5)	3116.8
**19**	2877.5	2879.3	960.9 (m/3), 721.0 (m/4), 577.1 (m/5)	2878.6
**FAM-19**	3193.6	3195.6	1066.5 (m/3), 800.02 (m/4), 640.4 (m/5)	3194.6
**20**	2941.6	2943.5	n.d.	2942.7
**FAM-20**	3257.7	3259.7	1088.0 (m/3), 816.2 (m/4)	n.d.
**21**	2884.6	2886.4	n.d.	2885.6
**FAM-21**	3200.7	3202.7	1069.0 (m/3), 801.8 (m/4), 641.7 (m/5)	3201.9
**22**	2788.5	2790.2	1395.9 (m/2), 931.1 (m/3), 698.6 (m/4)	2789.5
**FAM-22**	3104.6	3106.5	1036.3 (m/3), 777.6 (m/4), 622.2 (m/5)	3106.1
**23**	2794.6	2796.4	933.4 (m/3), 700.4 (m/4)	2796.8

Table S2 continued:

Compound	M_{exact} (calc) [Da]	M_W (calc) [Da]	ESI+ (LC-MS)	MALDI-ToF
			m/z (exp)	M_{exact} (exp) $[M+H]^+$
**FAM-23**	3111.7	3113.6	1038.7 (m/3), 779.3 (m/4), 623.8 (m/5)	3122.9
**24**	2873.6	2875.4	959.4 (m/3), 719.8 (m/4), 575.9 (m/5)	2874.6
**FAM-24**	3189.6	3191.6	1064.6 (m/3), 798.9 (m/4), 639.3 (m/5)	3190.9
**25**	2880.7	2882.5	1441.9 (m/2), 961.8 (m/3), 721.4 (m/4)	2881.7
**FAM-25**	3196.7	3198.7	1067.2 (m/3), 800.6 (m/4), 640.7 (m/5)	3198.1
**26**	2789.5	2791.2	931.3 (m/3), 698.8 (m/4)	2790.5
**27**	2796.6	2798.4	1399.9 (m/2), 933.7 (m/3), 700.6 (m/4)	3198.1
**28**	2874.6	2876.3	959.6 (m/3), 720.1 (m/4), 576.1 (m/5)	2875.7
**29**	2881.7	2883.5	962.1 (m/3), 721.9 (m/4), 577.6 (m/5)	2882.4
**30**	2732.5	2734.2	1368.0 (m/2), 912.4 (m/3)	2733.5
**FAM-30**	3048.5	3050.4	1525.9 (m/2), 1017.8 (m/3), 763.7 (m/4)	3050
**31**	2731.5	2733.2	1367.4 (m/2), 912.2 (m/3)	2732.4
**FAM-31**	3047.6	3049.5	1525.5 (m/2), 1017.4 (m/3), 763.4 (m/4)	3048.7
**32**	2774.6	2776.3	1388.9 (m/2), 926.4 (m/3)	2775.5
**FAM-32**	3090.6	3092.5	1547.0 (m/2), 1031.8 (m/3), 774.1 (m/4)	3092.2
**33**	2773.6	2775.3	1388.5 (m/2), 926.2 (m/3)	2774.5
**FAM-33**	3089.6	3091.5	1546.5 (m/2), 1031.5 (m/3), 773.9 (m/4)	3090.8
**34**	2838.7	2840.4	1421.1 (m/2), 947.7 (m/3)	2839.9
**FAM-34**	3154.7	3156.7	1579.0 (m/2), 1053.2 (m/3), 790.2 (m/4)	3156.1
**35**	2838.7	2840.4	1420.9 (m/2), 947.8 (m/3)	2839.5
**FAM-35**	3154.7	3156.7	1578.7 (m/2), 1053.4 (m/3)	3156.2
**36**	2881.7	2883.5	1442.5 (m/2), 962.2 (m/3)	2882.9
**FAM-36**	3197.7	3199.7	1600.8 (m/2), 1067.5 (m/3), 801.0 (m/4)	3199.1
**37**	2853.6	2855.4	1428.5 (m/2), 952.8 (m/3)	2854.9
**FAM-37**	3169.7	3171.7	1058.074 (m/3), 793.9 (m/4)	3170.8

n.d.: not determined

3. Thermal shift assay

Table S3. Melting temperatures (T_m) of free Doc_{STm} toxin at 5 µM and Doc_{STm} (5 uM) in the presence of 50 µM of Phd^{52-73} peptides **1-37**. For the calculation of thermal shifts ΔT_m, the average T_m of free Doc_{STm} toxin determined in the respective peptide measurement cycles was subtracted from the average T_m of Doc_{STm} in the presence of peptide.

Peptide	Average T_m (°C)	Replicates	Doc_{STm} control Average T_m (°C)	Doc_{STm} control Replicates
**1**	71.0 ± 1.1	48	42.2 ± 2.4	50
**2**	66.4 ± 0.2	6	39.2 ± 1.3	6
**3**	67.4 ± 0.2	6	39.2 ± 1.3	6
**4**	56.8 ± 2.1	6	39.2 ± 1.3	6
**5**	69.7 ± 0.7	6	39.2 ± 1.3	6
**6**	67.3 ± 1.4	6	39.2 ± 1.3	6
**7**	70.2 ± 0.3	6	39.2 ± 1.3	6
**8**	67.8 ± 0.1	3	44.7 ± 0.2	3
**9**	72.3 ± 0.4	6	39.2 ± 1.3	6
**10**	59.5 ± 0.3	3	44.7 ± 0.2	3
**11**	73.5 ± 1.3	6	39.2 ± 1.3	6
**12**	68.3 ± 0.9	6	39.2 ± 1.3	6
**13**	72.7 ± 0.4	6	39.2 ± 1.3	6
**14**	56.0 ± 0.1	3	44.7 ± 0.2	3
**15**	57.8 ± 0.7	3	44.7 ± 0.2	3
**16**	65.0 ± 0.4	6	45.7 ± 0.4	6
**17**	61.3 ± 0.3	6	45.7 ± 0.4	6
**18**	66.3 ± 0.2	6	45.7 ± 0.4	6
**19**	62.5 ± 0.9	6	45.7 ± 0.4	6
**20**	60.4 ± 0.4	6	45.7 ± 0.4	6
**21**	61.1 ± 2.5	3	45.7 ± 0.4	6
**22**	54.7 ± 3.1	12	42.2 ± 0.8	11
**23**	57.5 ± 0.9	9	42.6 ± 0.5	8
**24**	54.3 ± 1.6	9	42.6 ± 0.5	8
**25**	56.4 ± 3.0	9	42.6 ± 0.5	8
**26**	62.9 ± 0.3	9	41.7 ± 0.5	8
**27**	61.8 ± 0.4	6	41.1 ± 1.1	6
**28**	62.1 ± 0.2	9	41.7 ± 0.5	8

Table S3 continued:

Peptide	Average T_m (°C)	Replicates	Doc_{STm} control Average T_m (°C)	Doc_{STm} control Replicates
**29**	61.6 ± 0.3	6	41.1 ± 1.1	6
**30**	56.7 ± 2.6	8	42.3 ± 0.4	7
**31**	61.6 ± 1.9	5	42.1 ± 0.2	5
**32**	62.6 ± 1.2	6	43.2 ± 1.0	5
**33**	63.0 ± 0.8	15	42.8 ± 0.8	14
**34**	58.9 ± 1.1	9	40.5 ± 3.1	9
**35**	59.9 ± 0.4	9	40.5 ± 3.1	9
**36**	61.3 ± 1.1	9	40.5 ± 3.1	9
**37**	55.3 ± 1.5	9	40.5 ± 3.1	9

4. Salmonella growth rescue experiment

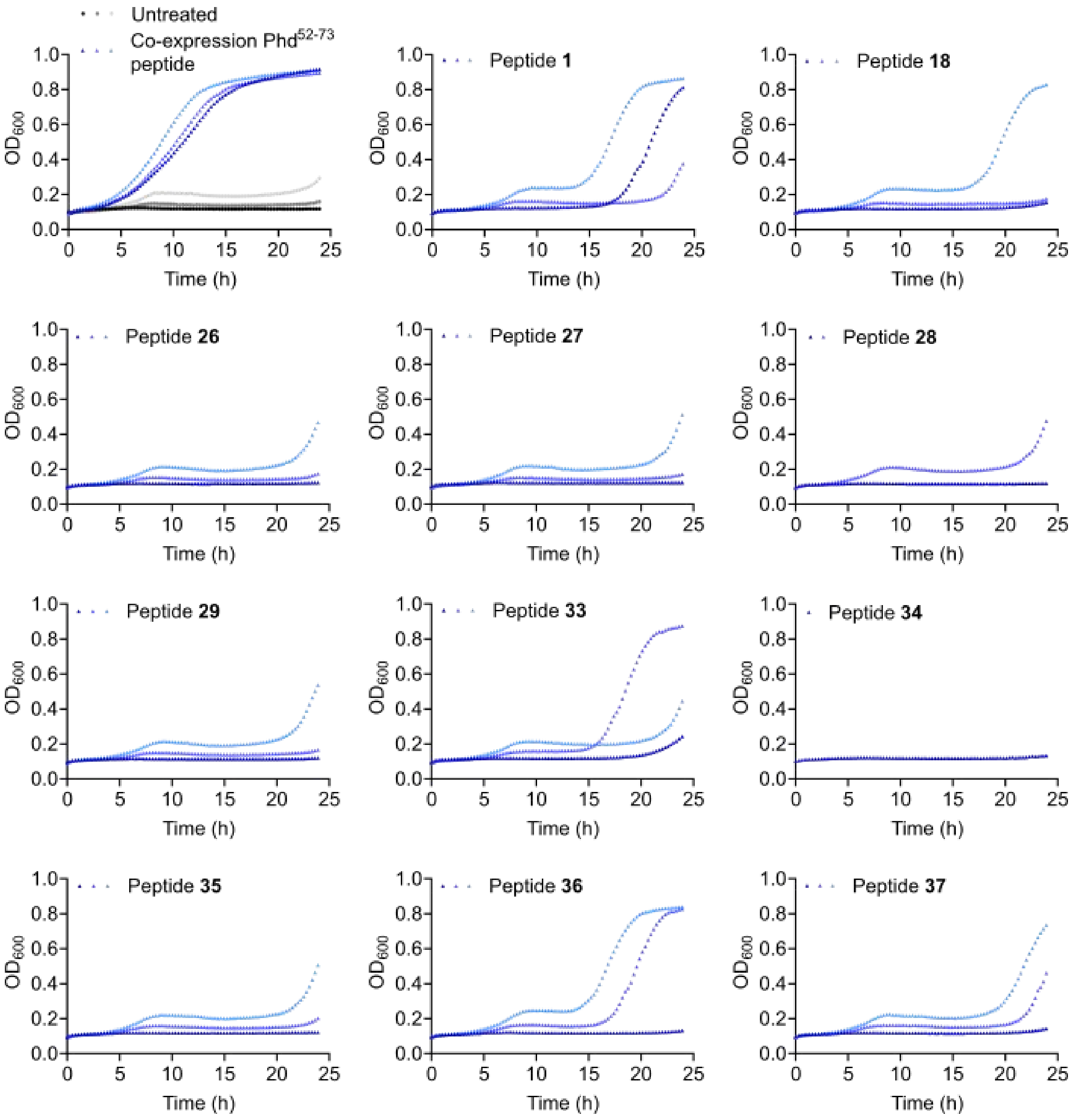

Figure S2. Growth curves of untreated Doc$_{STm}$-expressing *S.* Typhimurium (black/grey) and *S.* Typhimurium co-expressing Doc$_{STm}$ and Phd$_{STm}^{52-73}$ peptide (blue) from three independent experiments (Figure top left) as well as growth curves of Doc$_{STm}$-expressing *S.* Typhimurium treated with **2 µM** of peptide **1**, **18**, **26**, **27**, **28**, **29**, **33**, **34**, **35**, **36** or **37** from three independent experiments.

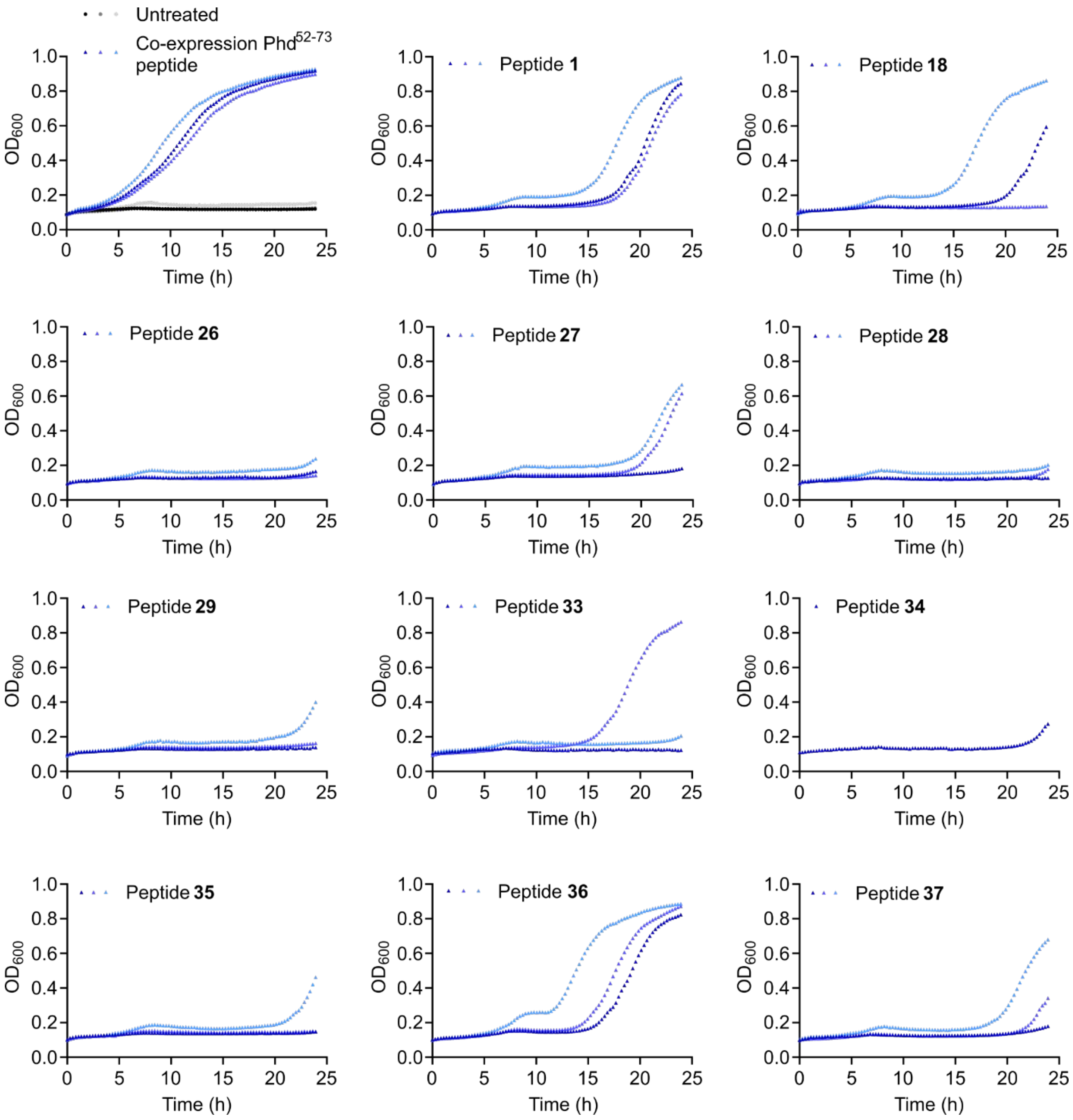

Figure S3. Growth curves of untreated Doc$_{STm}$-expressing *S.* Typhimurium (black/grey) and *S.* Typhimurium co-expressing Doc$_{STm}$ and Phd$_{STm}^{52\text{-}73}$ peptide (blue) from three independent experiments (Figure top left) as well as growth curves of Doc$_{STm}$-expressing *S.* Typhimurium treated with **10 µM** of peptide **1**, **18**, **26**, **27**, **28**, **29**, **33**, **34**, **35**, **36** or **37** from three independent experiments.

5. EF-Tu phosphorylation assay

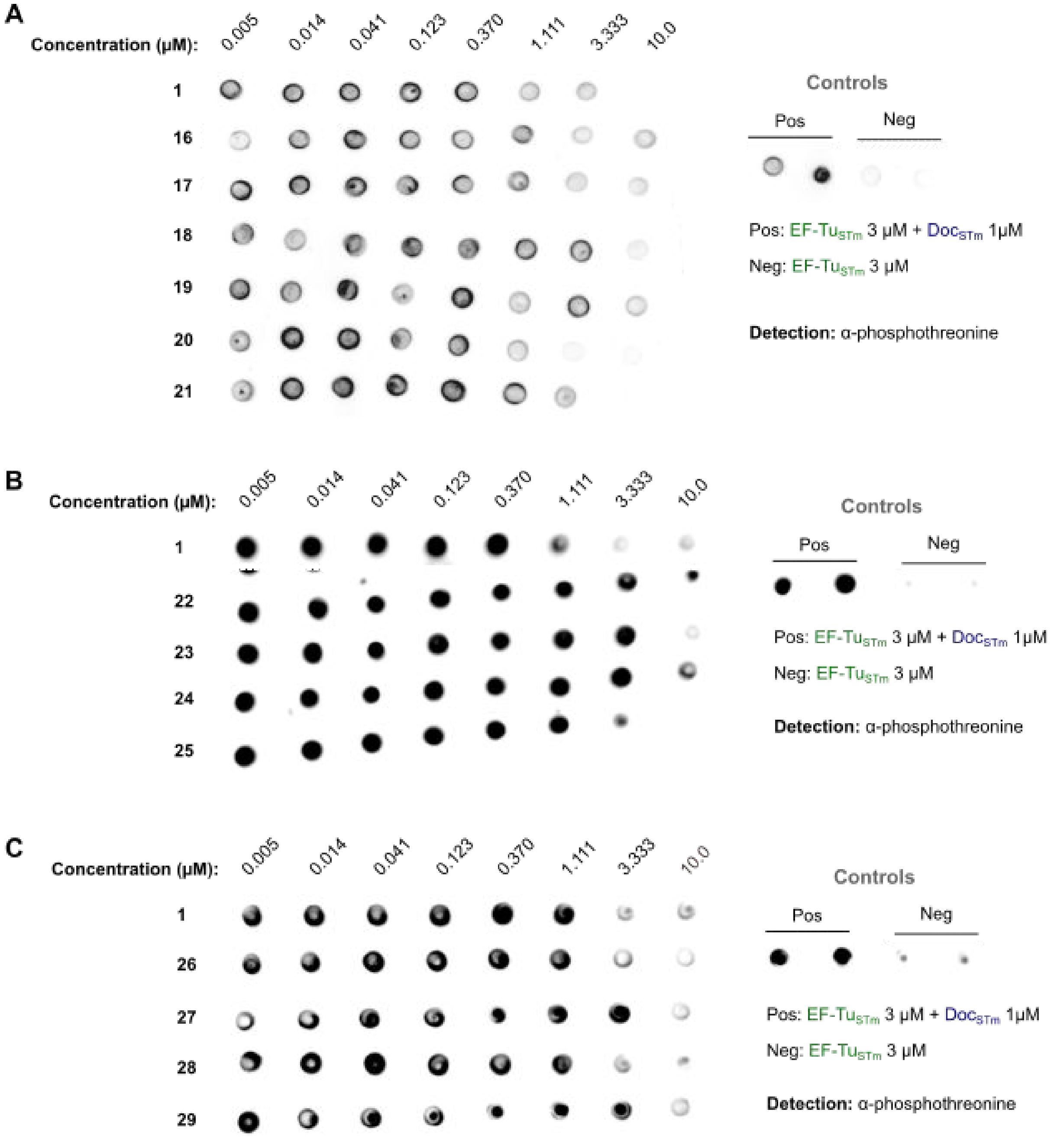

Figure S4. Replicates of dot blot phosphorylation assay with peptides 16-29. Dot blot detection of phosphorylated EF-Tu$_{STm}$ in the presence of Doc$_{STm}$ and Phd$^{52-73}$ peptide **1** as well as peptides **16-21 (A)**, **22-25 (B)** and **26-29 (C)**. Peptides were tested at eight concentrations, ranging from 10 µM to 5 nM (3-fold dilutions). Negative (EF-Tu$_{STm}$ 3 µM) and positive (EF-Tu$_{STm}$ 3 µM + Doc$_{STm}$ 1 µM) phosphorylation controls of the assays are shown on the right.

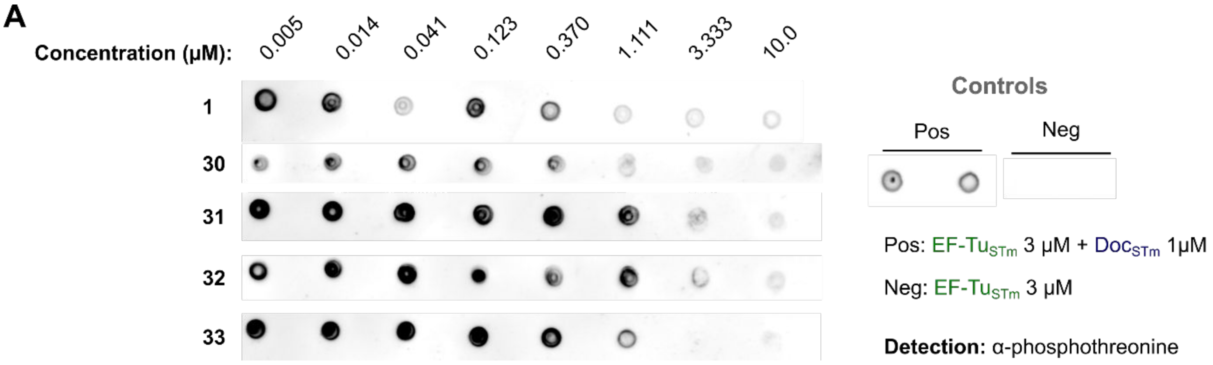

Figure S5. Replicates of dot blot phosphorylation assay with peptides 30-33. Dot blot detection of phosphorylated EF-Tu$_{STm}$ in the presence of Doc$_{STm}$ and Phd$^{52-73}$ peptide **1** and peptides **30-33**. Peptides were tested at eight concentrations, ranging from 10 µM to 5 nM (3-fold dilutions). Negative (EF-Tu$_{STm}$ 3 µM) and positive (EF-Tu$_{STm}$ 3 µM + Doc$_{STm}$ 1 µM) phosphorylation controls of the assay are shown on the right.

6. Online data repository

7. **Table S4.** DOIs for the individual folders for access to data for each peptide or individual experiments online data repository (DOI: 10.14469/hpc/12739).

Collection Name	DOI
Peptide **1**	10.14469/hpc/12740
Peptide **2**	10.14469/hpc/12741
Peptide **3**	10.14469/hpc/12742
Peptide **4**	10.14469/hpc/12743
Peptide **5**	10.14469/hpc/12744
Peptide **6**	10.14469/hpc/12745
Peptide **7**	10.14469/hpc/12746
Peptide **8**	10.14469/hpc/12747
Peptide **9**	10.14469/hpc/12748
Peptide **10**	10.14469/hpc/12749
Peptide **11**	10.14469/hpc/12750
Peptide **12**	10.14469/hpc/12751
Peptide **13**	10.14469/hpc/12752
Peptide **14**	10.14469/hpc/12753
Peptide **15**	10.14469/hpc/12754
Peptide **16**	10.14469/hpc/12755
Peptide **17**	10.14469/hpc/12756
Peptide **18**	10.14469/hpc/12757
Peptide **19**	10.14469/hpc/12758
Peptide **20**	10.14469/hpc/12759
Peptide **21**	10.14469/hpc/12786
Peptide **22**	10.14469/hpc/12787
Peptide **23**	10.14469/hpc/12788
Peptide **24**	10.14469/hpc/12789
Peptide **25**	10.14469/hpc/12790
Peptide **26**	10.14469/hpc/12791
Peptide **27**	10.14469/hpc/12792
Peptide **28**	10.14469/hpc/12793
Peptide **29**	10.14469/hpc/12794

Peptide **30**	10.14469/hpc/12795
Peptide **31**	10.14469/hpc/12796
Peptide **32**	10.14469/hpc/12797
Peptide **33**	10.14469/hpc/12798
Peptide **34**	10.14469/hpc/12799
Peptide **35**	10.14469/hpc/12800
Peptide **36**	10.14469/hpc/12801
Peptide **37**	10.14469/hpc/12802
Thermal Shift Data	10.14469/hpc/12803
Growth Rescue Experiments	10.14469/hpc/12804
Peptide synthesis codes	10.14469/hpc/12805
